# Disentangling motor planning and motor execution in unmedicated de novo Parkinson's disease patients: An fMRI study

**DOI:** 10.1016/j.nicl.2019.101784

**Published:** 2019-03-19

**Authors:** Jason A. Martin, Nadine Zimmermann, Lukas Scheef, Jakob Jankowski, Sebastian Paus, Hans H. Schild, Thomas Klockgether, Henning Boecker

**Affiliations:** a Functional Neuroimaging Group, Department of Radiology, University Hospital Bonn, Sigmund-Freud-Str. 25, 53127 Bonn, Germany; b Department of Neurology, University Hospital Bonn, Sigmund-Freud-Str. 25, 53127 Bonn, Germany; c Deutsches Zentrum für Neurodegenerative Erkrankungen (DZNE), University Hospital Bonn, Sigmund-Freud-Str. 25, 53127 Bonn, Germany; d Department of Radiology, University Hospital Bonn, Sigmund-Freud-Str. 25, 53127 Bonn, Germany

**Keywords:** Parkinson's disease, Self-initiated movement, Functional MRI, Motor networks, Compensation, Basal ganglia, BDI, Becks depression inventory, CSF, cerebral spinal fluid, DLPFC, dorsolateral prefrontal cortex, EHI, Edinburgh Handedness, ERROR, number of errors, fMRI, functional magnetic resonance imaging, FREE, self-initiated, FWE, family wise error correction, FWEc, family wise error with cluster correction, INSTRUCT, instruction condition, M1, primary motor cortex, MNI, Montreal Neurological Institute, MOTOR, movement execution, PET, positron emission tomography, PLAN, movement planning, PMC, premotor cortex, pre-SMA, pre-supplementary motor area, PSC, percentage-signal-change, REACT, externally triggered, REST, resting condition, ROI, region of interest, RT, reaction time, SMA, supplementary motor area, SNc, substantia nigra pars compacta, SPM, Statistical Parametric Mapping, STN, subthalamic nucleus, UPDRS, unified Parkinson's disease rating scale, WM, white matter

## Abstract

Many studies have used functional magnetic resonance imaging to unravel the neuronal underpinnings of motor system abnormalities in Parkinson's disease, indicating functional inhibition at the level of basal ganglia-thalamo-cortical motor networks. The study aim was to extend the characterization of functional motor changes in Parkinson's Disease by dissociating between two phases of action (i.e. motor planning and motor execution) during an automated unilateral finger movement sequence with the left and right hand, separately. In essence, we wished to identify neuronal dysfunction and potential neuronal compensation before (planning) and during (execution) automated sequential motor behavior in unmedicated early stage Parkinson's Disease patients. Twenty-two Parkinson's Disease patients (14 males; 53 ± 11 years; Hoehn and Yahr score 1.4 ± 0.6; UPDRS (part 3) motor score 16 ± 6) and 22 healthy controls (14 males; 49 ± 12 years) performed a pre-learnt four finger sequence (index, ring, middle and little finger, in order), either self-initiated (*FREE*) or externally triggered (*REACT*), within an 8-second time window. Findings were most pronounced during *FREE* with the clinically most affected side, where motor execution revealed significant underactivity of contralateral primary motor cortex, contralateral posterior putamen (sensorimotor territory), ipsilateral anterior cerebellum / cerebellar vermis, along with underactivity in supplementary motor area (based on ROI analyses only), corroborating previous findings in Parkinson's Disease. During motor planning, Parkinson's Disease patients showed a significant relative overactivity in dorsolateral prefrontal cortex (DLPFC), suggesting a compensatory overactivity. To a variable extent this relative overactivity in the DLPFC went along with a relative overactivity in the precuneus and the ipsilateral anterior cerebellum/cerebellar vermis Our study illustrates that a refined view of disturbances in motor function and compensatory processes can be gained from experimental designs that try to dissociate motor planning from motor execution, emphasizing that compensatory mechanisms are triggered in Parkinson's Disease when voluntary movements are conceptualized for action.

## Highlights

• Dissociated activations in early stage PD for motor planning and motor execution
• PD patients show frontal-parietal network compensation during self-initiated movement.
• Compensation for an impaired basal ganglia-premotor circuit occurs during planning.

## 1 Introduction

Parkinson's disease is a progressive neurodegenerative disorder caused by loss of striatal dopaminergic projections originating from the substantia nigra (Lees et al., 2009). Cardinal motor symptoms in Parkinson's disease include rigidity, tremor, and hypokinesia/akinesia, the latter being a characteristic inability to properly initiate movements at precise temporal and spatial scales that is thought to result from disruptions of distinct basal ganglia-premotor circuitries (Marsden, 1989): The basal ganglia segregate into limbic, associative, and motor domains along a rostro-caudal gradient (Avecillas-Chasin et al., 2016; Jung et al., 2014) and basal ganglia thalamo-cortical networks involving the associative and the motor domain of the basal ganglia play a distinct role for adequate planning and execution of motor tasks, respectively (Boecker et al., 2008; Jankowski et al., 2013). Impairment in the ability to initiate movements in Parkinson's disease is generally accentuated in association with increased cognitive load (Pieruccini-Faria et al., 2014, Pieruccini-Faria et al., 2016) and is particularly severe for complex motor tasks that require selective initiation of individuated finger movements within a precise temporo-spatial context along with integration of sensorimotor information (Benecke et al., 1987). Indeed, behavioral impairments in Parkinson's disease have been reported for simultaneous (Benecke et al., 1986) and sequential movements (Marsden, 1987), sequential cognitive task performance (Avanzino et al., 2013; Fama and Sullivan, 2002; Georgiou et al., 1994; Harrington and Haaland, 1991), memory for temporal sequence order (Vriezen and Moscovitch, 1990), and sequential learning (Doyon, 2008; Mochizuki-Kawai et al., 2010; Tremblay et al., 2010). Evidence in Parkinson's disease for hypokinesia/akinesia being a dopamine-dependent motor deficit manifesting already during conceptualization and planning of forthcoming actions has been provided by Avanzino et al., whom tested motor timing by using an established synchronization-continuation paradigm (Avanzino et al., 2013). Parkinson's disease patients and controls had to tap in synch to the tone of a metronome. After the tone stopped, participants had to either continue tapping with the same rhythm or to imagine tapping with the same rhythm. Deficits in the time reproduction task were found in Parkinson's disease patients in both continuation tasks, suggesting a selective motor sequence impairment due to a deficit in motor planning (Avanzino et al., 2013). Likewise, serial ordering deficits have been reported in early stages of Parkinson's disease (Ma et al., 2018).

Neuroimaging research in Parkinson's Disease with either positron emission tomography (PET) or functional magnetic resonance imaging (fMRI) has extensively studied the role of the basal ganglia thalamo-cortical motor loops in various motor tasks, however, hitherto without disentangling motor planning and motor execution. A recent meta-analysis using data from 283 Parkinson's disease patients taken from 24 functional neuroimaging studies reported differences in cortical activation during motor execution between Parkinson's disease patients and healthy controls in a left-lateralized fronto-parietal network (pre-supplementary motor area (pre-SMA), primary motor cortex (M1), inferior parietal cortex, and superior parietal lobule), and a consistent underactivity of the contralateral putamen (Herz et al., 2014). Attempts to delineate activation changes (i.e. activation decreases reflecting network impairments or activation increases reflecting compensatory mechanisms) that are specific to the conceptualization and planning of forthcoming motor actions are underrepresented in the literature. Some studies have used motor imagination to study the planning aspect of motor control in Parkinson's disease and initial PET activation studies of imagined joystick movements suggested abnormal cortical brain activation particularly contralateral to the clinically most affected side (Thobois et al., 2000). Relative reductions in cortical activation were reported in dorsolateral and mesial frontal areas during motor imagination in Parkinson's disease patients (Samuel et al., 2001), suggesting a dysfunction of cortical areas during motor preparation, a finding further substantiated in Parkinson's disease patients during OFF medication states (Cunnington et al., 2001). It has been suggested that such deficits in motor planning can be compensated by an increased reliance on visual information during the generation of motors plans. Impaired motor imagery in Parkinson's disease patients was found to be associated with an increased dependence on visual information (Helmich et al., 2007). These and other motor imagery studies (Maillet et al., 2015) support the notion of a disturbance in higher order motor control mechanisms in Parkinson's disease patients. That said, questions about the validity of motor imagery studies to properly capture motor planning processes still remain due to the inherent difficulties and limitations that may confound results. For example, controlling participant's compliance and strategy employed (i.e. the use of internal versus external imagery perspectives) can be difficult, particularly if participants cannot effectively use imagery (Dickstein and Deutsch, 2007; Mahoney and Avener, 1977). One further issue is that motor imagination studies involve the necessary suppression of voluntary motor output, a behavior that may lead to findings dominated by motor inhibition rather than representing motor planning per se.

In this study, we aimed to extend the characterization of motor-related changes in Parkinson's disease, with a specific focus on the conceptualization and planning phase of a forthcoming movement. We employed a sequential motor task that allows the disentanglement of motor planning and motor execution (Boecker et al., 2008; Jankowski et al., 2013), i.e. separately measuring the neural activity during the planning and execution of a pre-learnt sequence of finger movements, either self-initiated (*FREE*) or externally triggered (*REACT*) with either the affected or the non-affected hand. In accordance with a recent meta-analysis on motor tasks in Parkinson's disease (Herz et al., 2014), we hypothesized activation deficits in fronto-striatal and parietal brain regions, and notably in the posterior putamen contralateral to the performing hand (Herz et al., 2014). While primary sensorimotor / caudal SMA and caudal basal ganglia (motor domain: posterior putamen) activation deficits were expected during the motor execution phase, relative compensatory overactivity was expected during the motor planning phase in prefrontal cortical and rostral basal ganglia (associative domain: caudate nucleus, anterior putamen) areas. Compensatory mechanisms were expected to readily occur in the planning phase, in which movement patterns are conceptualized and the precise timings of subsequent motor actions are prepared for execution, necessitating higher effort in Parkinson's disease patients to adequately perform the complex task.

## 2 Material and methods

### 2.1 Participants

Twenty-two de novo Parkinson's disease patients (14 males; age 52.5 ± 10.7; range: 37–71 years) were included in this study. Patient evaluation of disease history and a full neurological examination for the diagnosis of idiopathic Parkinson's disease was performed in the Department of Neurology, University Hospital Bonn. Most patients presented with predominantly lateralized motor symptoms, 14 left hand side affected (see Table 1 for more detail), with an average unified Parkinson's disease rating scale (UPDRS, (Lang and Fahn, 1989)) motor score (part 3) of 15.6 ± 6.4 (range 5–28)). The total UPDRS score was 19.6 ± 7.4 and disease stage according to the Hoehn and Yahr score was 1.4 ± 0.6 (Hoehn and Yahr, 1967). For comparison, 22 healthy control participants (14 males; age 48.5 ± 12.4; range 21–68 years) were recruited, with no history of neurological or psychiatric disease. Participants were recruited between January 2008 and May 2013. Previous therapy or anti-parkinsonian medication was considered an exclusion criterion. Importantly, all patients were unmedicated both prior to and during participation in this study. Characteristics for all participants are shown in Table 1, including the Edinburgh handedness inventory (EHI: Oldfield (1971)) and the Beck depression inventory (BDI) (Hautzinger, 1991; Hautzinger et al., 1994).

**Table 1:** Participant characteristics.

Controls					Patients											
No.	Gender	Age	EHI	BDI	Gender	Age	EHI	BDI	UPDRS-I	UPDRS-II	UPDRS-III (motor)	UPDRS total	Hoehn & Yahr Scale	PD-Type	Affected body side	DAT-Scan
**1**	F	48	85	4	M	38	100	3	2	7	14	23	1	Akinetic-rigid	Right	Left
2	M	38	100	---	F	48	100	4	0	5	15	20	1	Akinetic-rigid	Left	Right
3	M	68	70	3	M	63	82	4	---	---	18	18	2	Akinetic-rigid	Left	Right
**4**	M	57	50	0	M	62	80	6	2	7	14	23	2	Akinetic-rigid	Right	Left
5	M	61	100	4	M	58	90	6	7	6	15	28	1	Equivalent	Left	Right
6	F	49	45	4	F	54	80	5	0	8	12	20	1	Akinetic-rigid	Left	Right
**7**	M	58	35	5	F	60	79	0	0	5	7	12	1	Akinetic-rigid	Right	Left
8	M	63	100	0	M	38	88	20	---	---	14	14	1	Akinetic-rigid	Left	Right
9	M	38	100	0	F	46	100	13	2	6	24	32	2.5	Akinetic-rigid	Left	Right
**10**	F	30	75	5	M	67	100	0	0	2	5	7	1	Tremor-dominant	Right	Left
11	F	60	65	9	M	41	−11	5	---	---	26	26	2.5	Akinetic-rigid	Left	Right
**12**	M	43	100	0	F	45	75	3	1	6	13	20	1	Akinetic-rigid	Right	No Info
**13**	F	57	39	8	M	72	80	4	---	---	8	8	1	Tremor-dominant	Right	Left
14	M	53	80	0	F	44	89	5	---	---	28	28	2.5	Equivalent	Left	Right
15	F	21	67	6	M	65	90	5	0	6	22	28	2	Tremor-dominant	Left	No Info
16	M	53	85	16	F	42	100	13	1	10	17	28	1	Akinetic-rigid	Left	Right
17	M	54	83	10	M	61	84	0	1	2	13	16	1	Tremor-dominant	Left	Right
**18**	M	35	67	7	M	66	62	4	0	1	27	28	1.5	Tremor-dominant	Right	Left
19	F	29	58	5	M	63	68	2	0	0	18	18	1	Tremor-dominant	Left	Right
20	M	47	81	1	F	41	86	9	0	0	10	10	1	Tremor-dominant	Left	Right
21	M	46	100	1	M	49	83	3	0	1	12	13	1	Tremor-dominant	Left	Right
**22**	F	60	65	9	M	46	82	2	0	1	11	12	1	Tremor-dominant	Right	Left
	Mean	48.5	75.0	4.6		53.0	81.2	5.3	0.9	4.3	15.6	19.6	1.4			
	SD	12.4	20.9	4.2		10.7	23	4.8	1.7	3.1	6.4	7.4	0.6			

EHI: Edinburgh Handedness Inventory; BDI: Beck Depression Inventory; UPDRS-I: Unified Parkinson's Disease Rating Scale – Part 1; UPDRS-II: Unified Parkinson's Disease Rating Scale – Part 2; UPDRS-III: Unified Parkinson's Disease Rating Scale – Part 3 – Motor examination score; UPDRS total: UPDRS total score equals the sum parts 1–3. Hoehn and Yahr Scale (H&Y). Please note that missing value is indicated by a ‘---’.

Bold No. indicates Parkinson disease patients with right hand affected.

### 2.2 Protocol approval and patient consent

This study was approved by the local Ethics Committee at the University Hospital Bonn (Ethikkommission an der Medizinischen Fakultät der Rheinischen Friedrich-Wilhelm's Universität Bonn; Ethics ID: 207/06) in accordance with the national legislation and the Declaration of Helsinki. All individuals voluntarily gave written informed consent before study enrolment and participation.

### 2.3 Task instruction and training

The task was previously used in healthy participants (Boecker et al., 2008) and in patients with writer's cramp (Jankowski et al., 2013). In essence, the task consists of a fixed automated sequence of four button presses (index, ring, middle, and little finger) that have to be either self-initiated (FREE) within a specified time period, or initiated upon an external visual cue (REACT). Prior to scanning, all participants had to learn and practice the predefined four-finger sequence by completing a 20–30 minutes training session on a computer using the same software as used during fMRI. Training ended once a consistent trial performance was attained with a minimum number of errors (i.e. <5%). During the practice, particular attention was given to encourage participants to vary the time point of movement initiation for the FREE condition by using the full range of the initiation time period (i.e. minimum 0 s, maximum 8 s) and to deter a repetitive strategy for sequence initiation. Once positioned within the scanner, participants rehearsed the task once more for approximately 10 min before the fMRI experiment, so that they were able to perform the movement sequence in a fluent, consistent and comfortable pace within the scanner environment, without the need to execute the sequence as quickly as possible.

### 2.4 Experimental paradigm

**Fig. 1:**
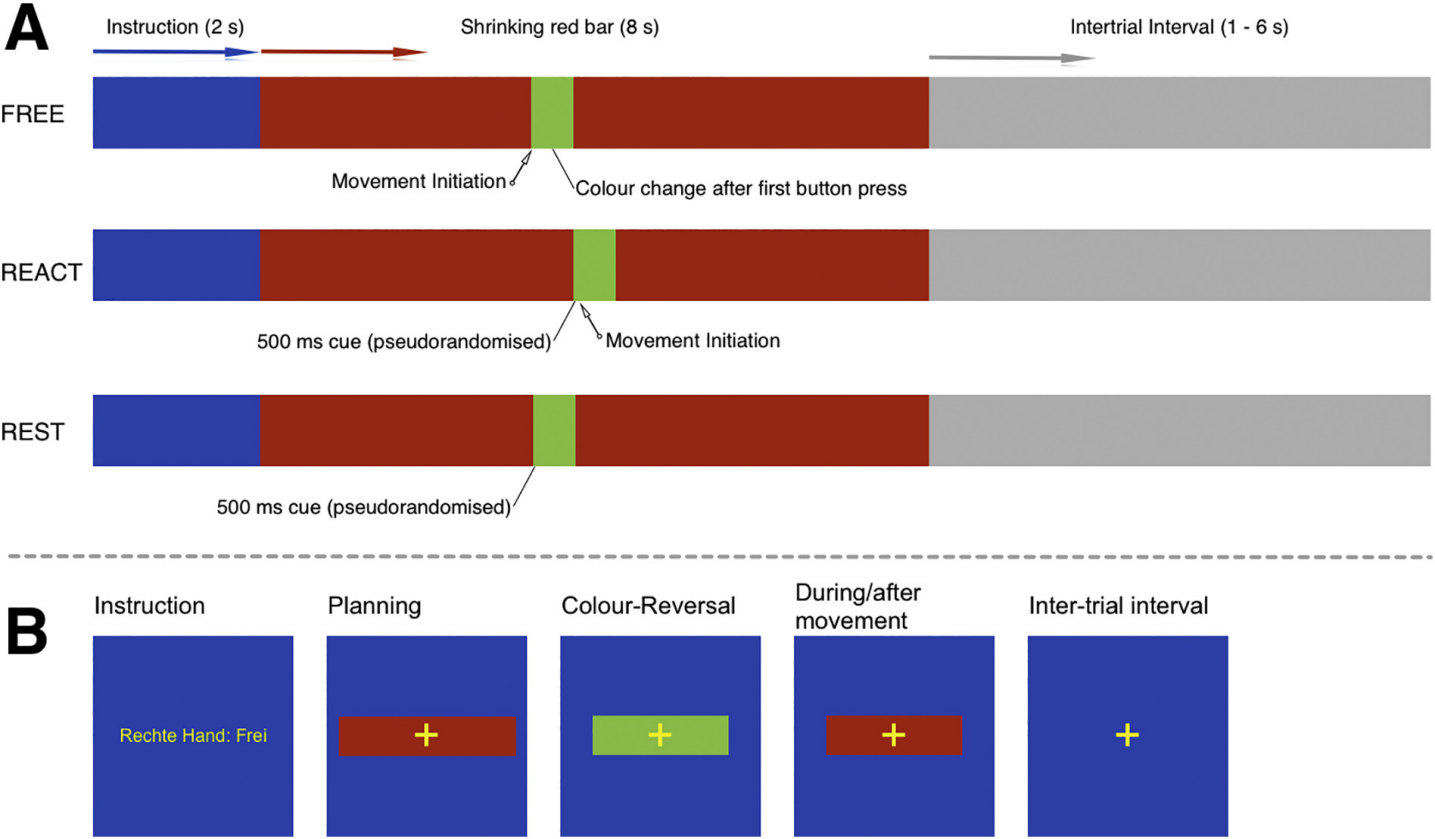
Experimental paradigm. (A) Schematic representation of the experimental paradigm (trial). *FREE* condition (upper line), movement onset triggers the color change in the visual stimuli (i.e. red bar to green cue). *REACT* condition (middle line), movement initiation is delayed with respect to the green visual cue. *REST* condition (lower bar), has no movement although the visual stimuli changes at pseudo randomized intervals in order to provide visual stimuli similar to *FREE* and *REACT*. (B) An example of how the different phases appeared visually to the participant.

The experimental paradigm consisted of three behavioral conditions: (1) FREE movement condition: where participants chose freely when to execute the predefined movement sequence, within the specified time frame; (2) REACT movement condition: where participants execute the predefined movement sequence in response to a visual cue; and (3) A rest condition (REST): where participants were instructed to passively view the visual stimuli and not move. Each movement condition was performed with either the left or right hand (L or R), per instruction. In more detail, each trial consisted of two parts (Fig. 1): (i) A visual instruction (2 s) that specified which of the five experimental conditions to perform (i.e. FREE-L, FREE-R, REACT-L, REACT-R, REST); ii) A time frame (8 s) during which the movement had to start, indicated on the visual display by a horizontally centered red bar that continuously shrank in size until it disappeared at the midpoint of the display. For FREE, the first button press induced an immediate color change of the horizontal bar to closely match the REACT visual input (i.e. red to green: 500 ms). For REACT, movement initiation was cued by a color change of the horizontal red bar (i.e. red to green, lasting 500 ms) that was programmed to appear at pseudo-randomized time points (mean 4.0 s, range 0.6–7.6 s after the horizontal red bar onset). For REST, participants were instructed to observe the horizontal bar and its randomized color changes without performing any movement. Each of the five experimental conditions occurred 30 times in a random order resulting in 150 trials. Each trial was separated by a variable inter-trial interval (mean duration 3.5 s, range 1.0–6.0 s). All button presses (i.e. finger movements) were recorded using two MR-compatible optical response keypads (LUMItouch, Photon Control Inc., Burnaby, Canada), to allow the precise timing of each button press to be recorded in a data log-file, which was used in the data analysis.

### 2.5 MRI procedures

The experiment was conducted using a 3 Tesla MRI scanner (either an Ingenia or an Achieva, Philips Medical Systems, Best, The Netherlands) equipped with an 8 channel SENSE head coil. Each experimental run consisted of 800 T2*-weighted gradient echo single-shot EPI volumes (spatial resolution: 3.6 × 3.6 × 3.6 mm^3^; 41 axial slices acquired in an interleaved ascending mode; TR = 2.595 s; TE = 35 ms, flip angle = 90°; FOV = 230 × 230 × 147.6 mm^3^) aligned approximately parallel to the anterior-to-posterior commissure line. For each participant, two anatomical images were collected using a high-resolution T1-weighted MPRAGE sequence (TR = 7.7 ms, TE = 3.9 ms, flip angle: 15°, spatial resolution: 1 mm^3^ isotropic; FOV: 256 × 256 × 180 mm^3^), and one with optimal quality selected for analysis. The total time for fMRI data acquisition was ~35 min. Visual stimuli were back-projected onto a semi-translucent screen outside the scanner using the Presentation software (Neurobehavioral Systems, Albany, CA, USA). Participants viewed the visual projection via a head coil mounted mirror. To minimize head movement and increase comfort, participants were provided with additional padding inside the head coil and further reminded to not make unnecessary movements other than those required to complete the experiment during scanning.

### 2.6 Data alignment according to clinically assessed affected side

As Parkinson's disease typically has an asymmetric clinical manifestation in early stages as tested here, the imaging and behavioral data of eight Parkinsonian patients with predominant basal ganglia pathology on the left side were transformed to bring about a uniform lateralization of the more affected and less affected sides in the entire cohort. This transformation was done by flipping the imaging data (i.e. left to right), prior to pre-processing, so that the most affected basal ganglia was always located in the right hemisphere. The side with the strongest deficit in presynaptic dopaminergic innervation was confirmed with DaTSCAN™ (GE Healthcare) single photon emission computed tomography (SPECT), except two patients with no DaTSCAN™ available. In these cases, the clinical diagnosis of the affected side was used as the only supporting rationale. Additionally, the same data transformation was applied to eight-corresponding matched healthy control participants to control any confounds due to the data treatment (e.g. differences in hand laterality). This transformation of the imaging data was accompanied with a likewise flip of the behavioral data. For clarity, throughout the remainder of this article, behaviors will be referred to as affected (i.e. more affected) and non-affected (i.e. less affected) rather than left and right hand. This approach was particularly relevant since all the de novo patients presented with unilateral symptoms at the time of study.

### 2.7 Analysis of behavioral data

To analyze the behavioral data, the following parameters were defined for each condition: Trial instruction (INSTRUCT) = time when the visual instruction was displayed; Movement planning time (PLAN) = time between the end of the visual instruction and the first button press; Motor execution (MOTOR) = the time between first and last button press for the finger sequence; and errors (ERROR). ERROR's were defined as any of the following: less or more than four button presses per motor sequence; wrong sequence of button presses; use of the wrong hand; button presses during REST; movement during non-movement phases; failure to execute movement. Note that PLAN and MOTOR were defined as independent behavioral events for each hand; also note that response times (RT) could only be evaluated in REACT and not in FREE (i.e. the time between the visual cue onset and the first button press) due to the participants' self-chosen movement initiation.

Behavioral data were statistically analyzed for differences between groups, conditions, and behavioral phases using repeated measures ANOVA's, with covariates for handedness and Parkinson's disease type included. Independent variables were group (Parkinson's disease patients, Controls), movement condition (FREE, REACT), action (PLAN, MOTOR), and hand (AFFECTED HAND, NON-AFFECTED HAND). Dependent variables were the durations or numerical assessments of the defined behaviors (PLAN, MOTOR, RT, and ERROR). Separate ANOVAs were performed to analyze differences between-groups and the within-subject factors ‘condition’, ‘action’ and ‘hand’, including possible interactions (e.g. hand x group; action x group; and hand x action x group, etc.), all tested for significant effects for PLAN, MOTOR, RT, and ERROR. Bonferroni-corrected post-hoc analyses were made where appropriate (significance level *p* < .05). Analyses were performed using SPSS v21 (Chicago, Illinois, USA).

### 2.8 fMRI data analysis

#### 2.8.1 Pre-processing and whole brain analysis

FMRI data analysis was done using the Statistical Parametric Mapping software (SPM12, Wellcome Department of Imaging Neuroscience, London, UK; http://www.fil.ion.ucl.ac.uk) implemented in MATLAB 7.5 (MathWorks). Prior to slice-timing correction and data realignment, the EPI images and anatomical T1 volume of eight Parkinson's disease patients and corresponding eight healthy control participants (indicated above; see Table 1) were flipped left to right using the reorient images utility within SPM12. Subsequently, slice-timing correction was applied (Sladky et al., 2011), after which all functional images of each participant were realigned and unwarped to the first image to correct for head movements; the anatomical T1 volume was then co-registered to the mean EPI image. After which the anatomical T1 was co-registered with the MNI-T1 template, and the same manipulation applied to all functional images; next the T1 scan was segmented and spatially normalized to match the MNI-T1 template provided by SPM12 using the unified segmentation and normalization procedure (Ashburner and Friston, 2005). The resulting transformation parameters were applied to the realigned functional images. Normalized functional images were then smoothed with an isotropic 8 mm full width at half maximum (FWHM) Gaussian kernel. At the first-level, analysis of individual participants' imaging data included the removal of low-frequency signal drifts using a high pass filter (cut off period 128 s) and a correction for temporal autocorrelation in the data applied using an autoregressive AR (1) process. Using the timings from the visual presentation and the recorded behavioral data from the LUMItouch keypad log-file, a vector per task condition was created that defined each behavioral event, specifying an onset and duration per event.

Behaviorally the events INSTRUCT, PLAN and MOTOR were defined per condition, and additionally REST. INSTRUCT was defined and modeled by specifying each epoch using the onset of visual presentation and a 2 s duration. PLAN and MOTOR were behaviorally defined events modeled by specifying the exact onsets and durations (See Fig. 1 and the behavioral parameters in Table 2). MOTOR duration was based on the keypad responses allowing a proper account of the end of PLAN and the start and end of MOTOR phases. These event-time vectors were used as the input for the 1st-level design specification that resulted in ten regressors of interest (i.e. INSTRUCT; 4 × PLAN and 4 × MOTOR: *FREE*_NON-AFFECTED HAND_, *FREE*_AFFECTED HAND_, *REACT*_NON-AFFECTED HAND_, *REACT*_AFFECTED HAND_, and 1 × REST), thereby, creating a boxcar function that specified the onset and duration of neural activity correlating with each event. REST was defined and modeled by specifying the onset time and an 8 s duration. This boxcar function was convolved with the default hemodynamic response function (HRF) implemented in SPM to create a time vector that predicts the time course of expected HRF based on the observed behaviors (Henson, 2004), with predictor functions created for all conditions. Also included within the model design were ERROR events and six covariates to capture residual movement-related artifacts (three rigid body translations and three rigid body rotations determined during realignment). To account for non-blood‑oxygen-level dependent specific signal inhomogeneities, we included the time course of the average signal from the white matter (WM) and cerebrospinal fluid (CSF) as additional nuisance covariate (Martin et al., 2015). To do this, we thresholded the individual WM and CSF masks produced by the T1 scan segmentation procedure with a probability value of 0.99 and then read out the mean time course signal within the WM and CSF volume from the realigned and normalized functional images using the MarsBar toolbox (http://marsbar.sourceforge.net) (Brett et al., 2002). After estimation of the 1st level general linear model (GLM), specific effects of the experimental conditions within each participant were tested by applying linear contrasts to the parameter estimates of the events of interest.

**Table 2:** Behavioral data. A summary of the behavioral data and the results of the between group statistical analysis for each of the defined behavioral conditions.

Parameter	Condition	Patients		Controls		*p* Value
		Mean ± SD	(Range)	Mean ± SD	(Range)	
PLAN duration (s)	FREE – Non-affected	3.52 ± 1.31	(1.11–6.27)	4.04 ± 0.79	(2.47–5.82)	.12 (t(34) = −1.59)^b^
	FREE – Affected	3.13 ± 1.46	(0.83–6.09)	3.98 ± 0.86	(2.98–5.52)	.02 (t(34) = −2.37)^b^
Response Time (s)	REACT – Non-affected	0.51 ± 0.18	(0.32–1.18)	0.56 ± 0.23	(0.32–1.09)	.46 (t(42) = −0.75)^a^
	REACT – Affected	0.47 ± 0.14	(0.13–0.79)	0.56 ± 0.23	(0.32–1.08)	.13 (t(27) = −1.55)^b^
MOTOR duration (s)	FREE – Non-affected	1.77 ± 0.96	(0.76–5.56)	1.51 ± 0.41	(0.66–2.67)	.24 (t(42) = 1.19)^a^
	FREE – Affected	2.07 ± 0.97	(0.82–5.66)	1.52 ± 0.43	(0.63–2.71)	.02 (t(42) = 2.45)^a^
	REACT – Non-affected	1.59 ± 0.54	(0.71–3.28)	1.47 ± 0.39	(0.52–2.51)	.38 (t(42) = 0.89)^a^
	REACT – Affected	1.87 ± 0.64	(0.75–3.37)	1.49 ± 0.38	(0.53–2.59)	.02 (t(42) = 2.42)^a^
ERROR (No.)	Non-affected	5.64 ± 7.27	(0–24)	3.23 ± 3.70	(0−13)	.18 (t(31) = 1.38)^b^
	Affect	6.02 ± 7.18	(0−31)	3.07 ± 3.28	(0–16)	.09 (t(29) = 1.76)^b^
	REST	0.05 ± 0.21	(0–1)	0.18 ± 0.50	(0–2)	.31 (t(35) = −1.04)^b^

a Two-sided t-test of independent samples of equal variance (Levene-test *p* < .05).

b Two-sided t-test of independent samples of unequal variance (Levene-test *p* < .05).

#### 2.8.2 First level analysis

First-level linear behavioral contrasts were calculated comparing each regressor with the implicit baseline (i.e., those time periods that were not explicitly modeled and those during which no event occurred) by setting the regressors of interest to 1 and all other regressors to zero. Then, first level linear contrasts were calculated, comparing events of the different conditions with *REST* (e.g., *FREE*_PLAN AFFECTED HAND_ > *REST*, *FREE*_MOTOR AFFECTED HAND_ > *REST*, *REACT*_PLAN AFFECTED HAND_ > *REST*, etc.) by setting the regressors of interest to 1 and the regressor of *REST* to −1 and all other regressors to zero. These contrasts were used to do the second level analysis.

#### 2.8.3 Second level analysis

To explore the difference between the Parkinson's disease patients and controls, the first-level linear behavioral contrasts were taken to the second level where they were subjected to a between group analysis of variance (ANOVA, full factorial design in SPM12) with a single factor condition defined (*FREE*_PLAN_, *FREE*_MOTOR_, *REACT*_PLAN_, *REACT*_MOTOR_, etc. for the AFFECTED HAND and the NON- AFFECTED HAND, respectively) and covariates for handedness and Parkinson's disease type included (Edinburgh Handedness Inventory was entered as a percentage score and Parkinson's disease type was entered as numbers 1–4 (‘1’ Akinetic-rigid, ‘2’ Tremor-dominant, ‘3’ Equivalent, and ‘4’ control participant)). T contrasts were made for between group comparisons and reported using a family-wise error corrected threshold of *P*_FWE_ < 0.05 at the voxel-level. When no clusters were present at a statistical threshold of *P*_FWE_ < 0.05, a statistical threshold of *p* < .001 with whole brain FWE cluster level correction (i.e. FWEc), as specified by SPM, was considered.

Anatomical localizations of peak functional imaging activations were determined using the WFU pick atlas v3.0 (Maldjian et al., 2003, 2004) and the SPM Anatomy Toolbox v2.0 (Eickhoff et al., 2005, 2006, 2007). For the purpose of illustration and anatomical localization of the results, the functional image contrasts were overlaid on the MNI-152 template brain using MRIcron software (Release 2 May 2016, https://www.nitrc.org/projects/mricron/, Chris Rorden, University of South Carolina, USA). All imaging coordinates are reported in a standard stereotactic reference space (MNI, Montreal Neurological Institute).

#### 2.8.4 Regions of interest (ROI) analysis

In addition to the parametric whole brain level analyses, between group-analyses were performed using a region of interest (ROI) approach. The intention of the ROI analysis was to provide a more detailed investigation of the hypo- and hyper-activations identified in the parametric between group comparisons, allowing a more comprehensive comparison with existing findings in the motor imaging literature in Parkinson's disease. Sixteen separate ROIs were created by computing a spherical volume of interest with a 5 mm radius centered on activation peaks previously reported (Yu et al., 2007), using the MarsBar toolbox (http://marsbar.sourceforge.net; Brett et al. (2002)). The locations chosen are typical of those regions reported to show abnormal activation in Parkinson's disease and the application of respective ROI based analysis in our data allows a more direct comparison with previous work reporting deficits and compensatory mechanisms for the defective basal ganglia in Parkinson's disease (Yu et al., 2007). These regions were transformed from Talairach coordinates into MNI based coordinates using the appropriate ‘tal2mni’ MATLAB script (matched by reported software package) based on the findings of Lancaster et al. (2007) available for download from brainmap.org (http://www.brainmap.org/icbm2tal/). Right M1 had not been previously reported in the results of Yu et al., 2007, and so for simplicity the coordinates of the left M1 reported by Yu et al. were flipped left-to-right using SPM, visually verified for anatomical correctness and used as the ROI of right M1. ROIs identified were: left M1: −33, −18, +68; right M1: +36–18 +68; left pre-SMA: −9, +12, +50; right pre-SMA: +10, +12, +51; left SMA: −9, +5, +53; right SMA: +11, +2, +59; left DLPFC: −41, +49, +28; right DLPFC: +44, +50, +27; left caudate: −14, +6, +19; right caudate: +17, 0, +18; left putamen: −24, +1, +4; right putamen: +25, −6, +8; left globus pallidum (L-Gpe): −21, +3, 0; right globus pallidum (R-Gpe): +23, +3, −1; left cerebellum: −28, −70, −33; right cerebellum: +43, −60, −32). The MarsBar toolbox was used to extract and calculate the percentage-signal-change (averaged across all trials) within each sphere region for all respective events for each participant and the average calculated for each condition event. Percentage-signal-change (PSC) for all trials of a given condition were averaged within subjects. Individual PSC for each condition were finally averaged across each participant group.

## 3 Results

### 3.1 Behavioral data

All the recruited participants completed the task successfully. The behavioral results are summarized in Table 2, despite both groups having a similar success in task performance, statistical analysis revealed some between group differences: The ANOVA showed significant differences in ‘action’ (F (1,40) = 7.686, *p* = .008), where planning was significantly longer than motor execution in both groups. There was a significant ‘group × action’ interaction (F (1,40) = 4.350, *p* = .043), and a significant ‘group × action × hand’ interaction (F (1,40) = 6.535, *p* = .014). Post hoc *t*-tests (significance threshold *p* < .05) revealed, for control participants, that the hand used for task performance made no significant difference to either the planning time or motor execution time. Parkinson's disease patients had significantly shorter planning times than controls in the *FREE*_AFFECTED HAND_, and significantly longer motor execution time both during *FREE*_AFFECTED HAND_ and *REACT*_AFFECTED HAND_ compared to controls. Parkinson's disease patients showed a trend of slower task motor execution compared to controls (not significant) with the NON-AFFECTED HAND. Parkinson's disease patients made significantly more errors with the AFFECTED HAND compared to controls, along with a tendency for more errors with the NON-AFFECTED HAND. The ANOVA, for the behavioral data, showed no further significant differences for between-subject factors, within-subject factors, or interactions.

### 3.2 Functional MRI data

#### 3.2.1 Within-group comparisons

**Fig. 2:**
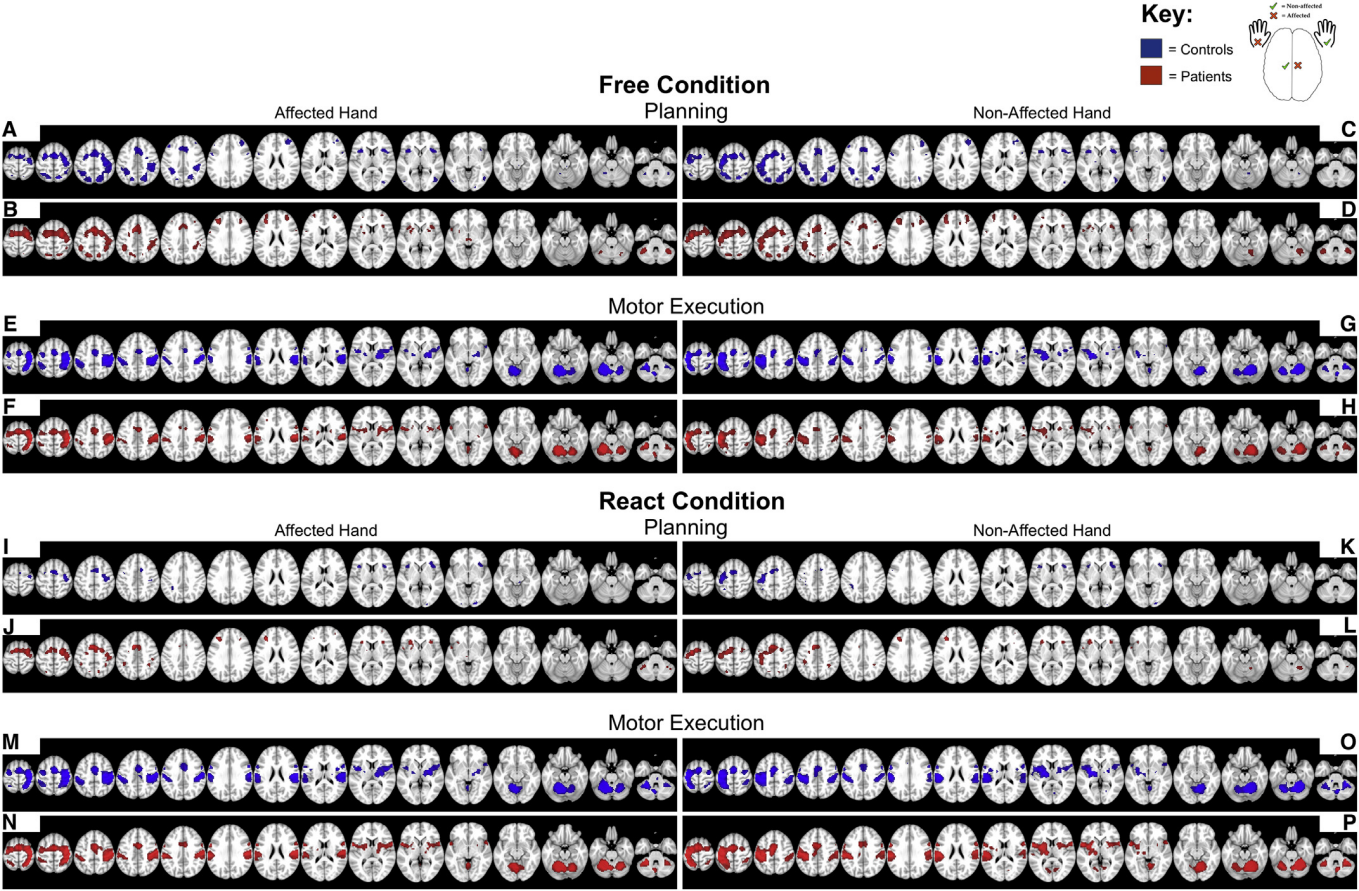
Within-group analysis. Within-group activations for the preparation and execution of action, compared to *REST*, in healthy control participants (Blue: A, C, E, G, I, K, M, O) and de novo Parkinson's Disease patients (Red: B, D, F, H, J, L, N, P). Activation for the preparation and motor execution of self-initiated action are shown in the top half of the figure (*FREE*_PLAN_ and *FREE*_MOTOR_, respectively). Activation for the preparation and motor execution of externally cued action are presented in the figure's lower half (*REACT*_PLAN_ and *REACT*_MOTOR_, respectively). Shown is the significant within-group signal increases (*p* < .05, FWE corrected at voxel-level) represented on an MNI-T1-template brain, displayed using multiple axial transections. Coordinates shown (z only) are in MNI-space. (For interpretation of the references to color in this figure legend, the reader is referred to the web version of this article.)

The data of the fMRI main effects comparison with REST showed that both groups (Fig. 2: Controls: A, C, E, G, I, K, M, O; Parkinson's disease patients: B, D, F, H, J, L, N, P) revealed a rostro-caudal shift in the center of mass of the activations from pre-SMA and lateral premotor cortices (PMC) during planning (Fig. 2: Controls: A, C, I, K; Parkinson's disease patients: B, D, J, L) to SMA proper and primary sensorimotor areas during motor execution (Fig. 2: Controls: E, G, M, O; Parkinson's disease patients: F, H, N, P), as would be expected based on previous research with this paradigm (Boecker et al., 2008; Jankowski et al., 2009, 2013). During *FREE*_PLAN_, activation of the dorsolateral prefrontal cortex occurred bilaterally in the Parkinson's disease group (Fig. 2B, D, J), whereas it tended to occur unilaterally in the controls (Fig. 2A, C). In the basal ganglia, variable rostral striatal activation was found during planning (particularly in Parkinson's disease patients, i.e. Fig. 2B, D, J; but also, in controls, i.e. Fig. 2C), and a consistent contralateral posterior striatal activation during motor execution, both in Parkinson's disease patients (Fig. 2F, H, N, P) and in controls (Fig. 2E, G, M, O). Both groups equally showed a larger involvement of the cerebellum during motor execution as compared to motor planning.

#### 3.2.2 Between-group comparisons

**Fig. 3:**
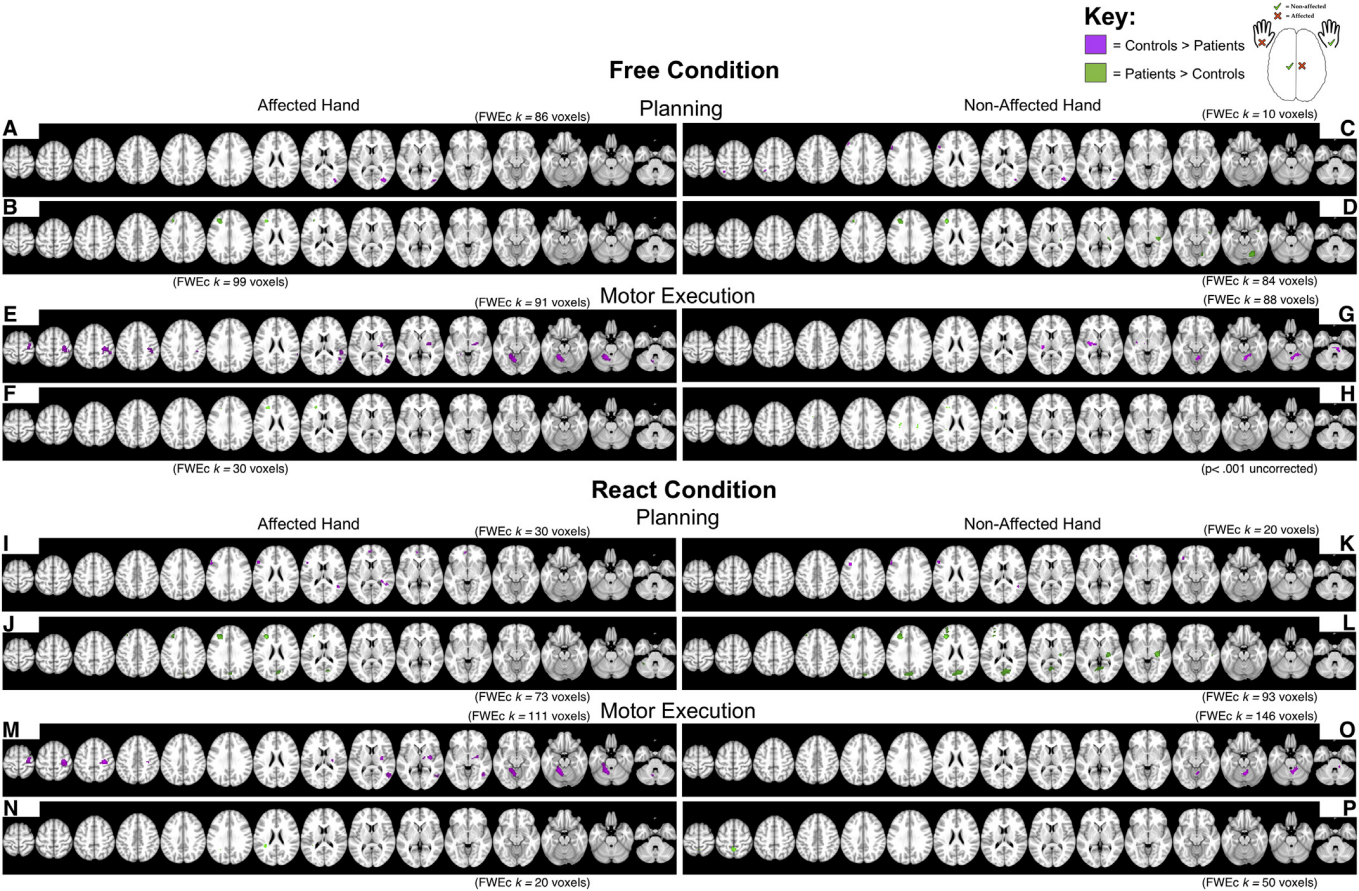
Between-group analysis. The outcome of the between-group comparisons for the preparation (PLAN) and the execution (MOTOR) of the movement sequence. Represented are the between-group activation comparisons during the planning during the self-initiated (*FREE*; A–D) and externally cued (*REACT*; I-L) action, along with the MOTOR between-group activation comparisons for the self-initiated (*FREE*; *E*-H) and externally cued (*REACT*; M-P) action. The activations are color coded for the comparison direction, Controls > Parkinson's disease Patients (in violet; A, C, E, G, I, K, M, O) and Parkinson's disease Patients > Controls (in green; B, D, F, H, J, L, N, P). Analyses shown are the significant between-group comparisons (the reported statistical threshold are either *p* < .001 uncorrected at the voxel-level or *p* < .001 uncorrected at the voxel-level with a cluster size correction (*k*, indicated; *p* < .05 corrected at the cluster-level: FWEc) represented on an MNI-T1-template brain, displayed using multiple axial transections. Coordinates shown (z only) are in MNI-space.

The relative differences in cortical activations between Parkinson's disease patients and controls were calculated for the conditions *FREE* and the *REACT* separately and were determined for the AFFECTED HAND and the NON-AFFECTED HAND, respectively (Fig. 3).

During motor execution, Parkinson's disease patients showed a consistent underactivity compared to controls in the contralateral posterior putamen in all movements with the AFFECTED HAND (Fig. 3E, M), but only partly with the NON-AFFECTED HAND (during *FREE*_MOTOR_ only; Fig. 3G). Moreover, Parkinson's disease patients showed a consistent underactivity in the ipsilateral anterior cerebellum / cerebellar vermis during motor execution (Fig. 3E, G, M, O). By appearance, the reported underactivity tended to be more profound and extent in movements with the AFFECTED HAND, relative to the NON-AFFECTED HAND. Additionally, motor execution with the AFFECTED HAND showed a consistent underactivity in the contralateral primary motor cortex in the Parkinson's disease group (Fig. 3E, M). No significant activation difference emerged during motor execution at the level of the SMA for the whole brain a comparison at the FWE corrected statistical threshold.

During *FREE*_PLAN_, Parkinson's disease patients showed a relative overactivity in the DLPFC ipsilateral to the AFFECTED HAND or the DLPFC contralateral to the NON-AFFECTED HAND (Fig. 3B, D). During *REACT*_PLAN_, Parkinson's disease patients also showed a relative overactivity in the DLPFC ipsilateral to the AFFECTED HAND and the DLPFC contralateral to the NON-AFFECTED HAND (Fig. 3J, L). To a variable extent this relative overactivity in the DLPFC went along with a relative overactivity in the precuneus during the *REACT*_PLAN_ conditions (Fig. 3J, L). Relative overactivity in PD patients at the level of the ipsilateral anterior cerebellum/cerebellar vermis was only observed in the *FREE*_PLAN_ (NON-AFFECTED HAND) (Fig. 3D).

#### 3.2.3 Region of interest analysis

For ROI analysis, the significance of the between group differences were estimated using non-parametric Mann-Whitney *U* tests for each condition separately (i.e. *FREE*_PLAN_, *FREE*_MOTOR_, *REACT*_PLAN_, *REACT*_MOTOR_). Regions that had a significant difference at *p* < .05 were identified, for each region, and listed in Table 3. We first describe the between group differences where de novo Parkinson's disease patients were hypoactive (relative under-activity) compared to controls. Next, we describe the hyperactive regions (relative over-activity) in de novo Parkinson's disease patients compared to controls.

**Table 3:** ROI analysis. Shows a summary of the between group analysis using data for each of the region of interest (ROI: i.e. percentage signal change). The significance differences were estimated using non-parametric Mann-Whitney *U* test for each behavioral condition separately (i.e. *FREE*_PLAN_, *FREE*_MOTOR_, *REACT*_PLAN_, *REACT*_MOTOR_), for each ROI separately.

Region	Hemisphere	Centre of mass in MNI coordinates			*FREE*_PLAN_	*FREE*_MOTOR_	*REACT*_PLAN_	*REACT*_MOTOR_
		X	Y	Z				
M1	L	−32.6	−17.7	68.3	0.237	0.398	0.730	0.916
	R	+36	−18	68	0.638	0.105	0.645	0.116
pre-SMA	L	−9.1	11.9	50.4	0.103	0.913	0.538	0.304
	R	10.4	12.1	51.2	0.604	0.336	0.629	0.186
SMA	L	−9.0	4.7	53.4	0.111	0.953	0.299	0.842
	R	11.6	2.1	58.9	0.068	0.706	0.029⁎	0.095
DLPFC	L	43.5	49.7	26.7	0.135	0.439	0.758	0.247
	R	−40.8	49.4	28.2	0.258	0.728	0.580	0.316
Caudate	L	−13.8	5.7	18.6	0.253	0.346	0.734	0.315
	R	16.5	0.4	17.5	0.745	0.385	0.718	0.440
Putamen	L	−24.8	1.0	3.6	0.346	0.724	0.554	0.879
	R	25.0	−5.8	7.9	0.043⁎	0.001⁎	0.033⁎	0.003⁎
Globus Pallidum	L	−20.5	2.9	0.0	0.906	0.991	0.991	0.459
	R	22.7	3.0	−0.8	0.128	0.301	0.289	0.452
Cerebellum	L	−28.3	−70.0	−33.0	0.048⁎	0.641	0.439	0.839
	R	43.0	−59.9	−31.9	0.333	0.915	0.766	0.972

⁎ Indicates that the difference is significant (*p* < .05).

**Fig. 4:**
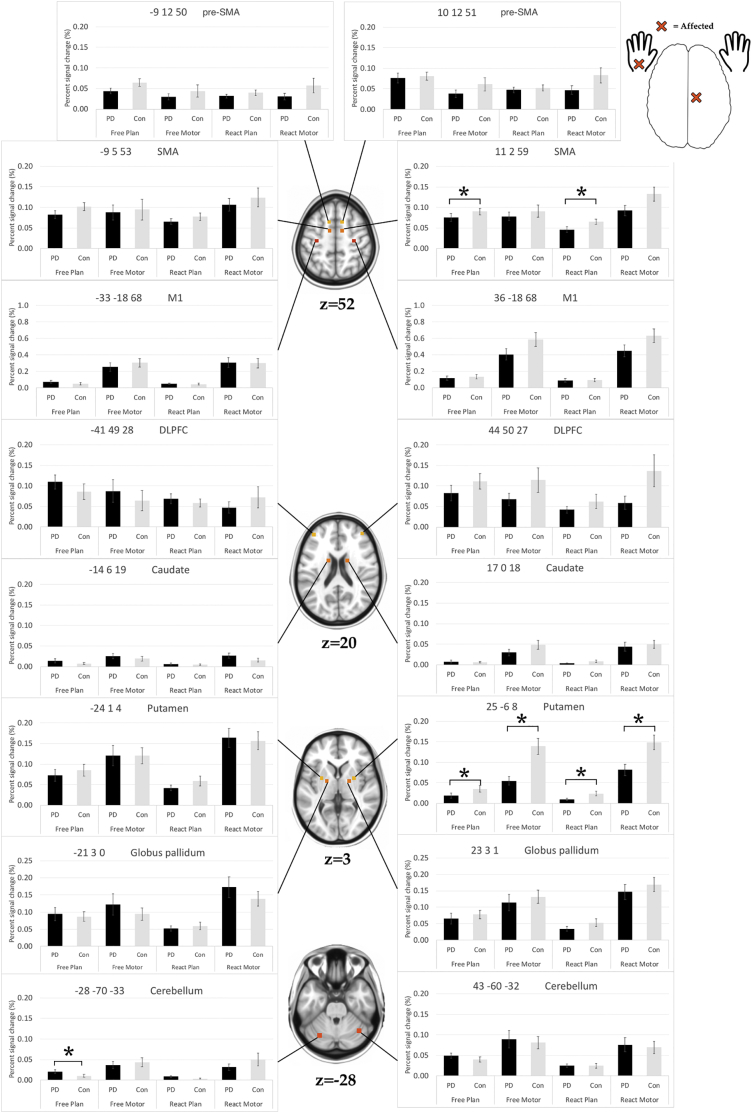
ROI analysis. Illustration of the location of the sixteen regions from the region of interest (ROI) analysis (bilateral – M1, SMA, pre-SMA, DLPFC, caudate, putamen, globus pallidum, and cerebellum) on a template. Data shown are group percentage-signal-change (mean ± SEM) for the affected hand: for each ROI, we extracted the beta estimate and calculated the percentage-signal-change for each participant and the average calculated for each condition event and averaged across participant group. The significance of the group differences was estimated using non-parametric Mann-Whitney *U* test for each condition separately (i.e. *FREE*_PLAN_, *FREE*_MOTOR_, *REACT*_PLAN_, *REACT*_MOTOR_). The asterisks indicate that the difference is significant (*p* < .05).

Parkinson's disease patients had significant underactivity in two regions (Table 3, Fig. 4), namely the SMA and the putamen contralateral to the AFFECTED HAND. In the SMA, which was generally more active during planning than execution, the percent signal change (contralateral to the AFFECTED HAND) was significantly lower in Parkinson's disease patients compared to healthy controls in both *FREE*_PLAN_, and *REACT*_PLAN._ In the putamen, which was generally more active during execution than planning, the percent signal change was significantly lower in Parkinson's disease patients compared to healthy controls in all conditions (i.e. *FREE*_PLAN_, *FREE*_MOTOR_, *REACT*_PLAN_, *REACT*_MOTOR_), i.e. Parkinson's disease patients exhibited a much lower signal for both planning and execution contralateral to the AFFECTED HAND. In sum, both the SMA (planning only) and putamen (planning and execution) in the hemisphere contralateral to the AFFECTED HAND showed to be hypoactive in de novo Parkinson's disease patients.

Parkinson's disease patients showed a significant overactivity in the lateral cerebellum ipsilateral to the AFFECTED HAND during *FREE*_PLAN_ (Table 3, Fig. 4).

## 4 Discussion

This study, to our knowledge, for the first time investigated defined stages of the motor cycle (i.e. the planning and the execution phases of automated sequential movements, either self-initiated or externally triggered with the affected and non-affected hands) in a well-defined cohort of unmedicated early stage Parkinson's disease patients. All data reported here were aligned on the basis of clinically determined basal ganglia pathology via DaTSCAN™ scans and/or clinical assessments, considering individual Parkinson's disease clinical phenotypes and handedness. Results are reported on the basis of parametric between-group analyses using stringent statistical criteria. Additional ROI analyses in prototypical areas derived from previous motor imaging research in Parkinson's disease provide measures of mean percent signal changes per area and condition in patients and controls, respectively, to allow comparisons with the published literature in this field of research.

Analyzing the different phases of the motor cycle in detail, we observed patterns of over- and under-activity in distinct brain areas that corroborate previous findings of abnormal processing during the motor execution phase, while highlighting potentially compensatory mechanisms during the planning phase of the subsequent motor actions. These results on the different phases of action help to better delineate and define the underlying mechanisms mediating motor sequence initiation in hypo-dopaminergic states. We will discuss our findings from both phases of action separately in the context of existing research data and pathophysiological concepts in Parkinson's disease.

### 4.1 Behavioral data

Behaviorally, both controls and Parkinson's disease patients showed a comparable task performance during the planning phase and the execution phase of self-initiated or externally triggered movements (Table 2). Parkinson's disease patients successfully performed the overlearned movement sequence with no apparent influence of hypokinesia or bradykinesia. Despite a successful motor performance notable, between group differences were apparent for the actions performed particularly with the affected hand. Parkinson's disease patients spent significantly less time planning movements in the *FREE* condition and responded significantly faster to the external visual cue during the *REACT* condition, compared to controls. During motor execution, Parkinson's disease patients had an overall tendency for a significantly slower motor execution during both, *FREE* and *REACT* with the affected hand only. Beyond these differences in time taken to perform the sequences, the overall similarity in task performance between groups is important to highlight, as it allows one to consider differences in activation to be representative of differences in system's function and potentially compensation, rather than being attributable to differences in the rate of movement or the duration of task performance per se (Deiber et al., 1999; Sadato et al., 1997).

### 4.2 fMRI data

Our fMRI main effects determined on within-group parametric analyses (Fig. 2) show that controls and Parkinson's disease patients recruited similar brain regions for the planning and the execution of the overlearned hand movement sequences, as would be expected based on previous reports with this motor task (Boecker et al., 2008; Jankowski et al., 2009, 2013). The main effects also highlight the well-established transition of activated brain areas characterizing motor planning and motor execution, as previously described with this paradigm in healthy subjects (Boecker et al., 2008). These transition patterns were also found in the Parkinson's disease patients. Between-group parametric analyses revealed distinct differences between the controls and the Parkinson's disease patients, that were in part confirmed by additional ROI analyses.

#### 4.2.1 Motor execution phase

In both groups and conditions, whole-brain parametric analyses revealed a fairly consistent pattern of activation (contralateral M1, SMA, contralateral posterior putamen, and ipsilateral > contralateral motor cerebellum) during the execution phase of the automated movement sequence. Nevertheless, on between-group analyses relative underactivity was seen in Parkinson's disease patients relative to controls in contralateral posterior putamen, contralateral M1, and ipsilateral anterior cerebellum/cerebellar vermis. These decreases in activation were particularly pronounced during performance with the affected hand. While the findings in putamen and M1 could be further corroborated using ROI analyses, the observation of a relative underactivity in the ipsilateral cerebellar vermis during execution of the affected and the non-affected hands in both conditions (i.e. during *FREE*_MOTOR_ and *REACT*_MOTOR_) was only found in the parametric analysis. We assume this to be due to the ROI location in a more posterior and lateral region of the cerebellum, as informed by previous published research. The reported between-group activation decreases corroborate previous findings in Parkinson's disease and suggest them to occur in early clinical disease stages, given that all individuals with Parkinson's disease were unmedicated de novo patients.

The functional underactivity apparent in the posterior putamen in movements with the affected hand (and less consistent with the non-affected hand) fits to our meta-analytically informed hypothesis (Herz et al., 2014). Demonstration of putamen underactivity already in unmedicated early stage Parkinson's disease, is highly compatible with the progressive loss of dopaminergic projection neurons from the substantia nigra pars compacta (SNc) to the basal ganglia, especially in the posterior *motor* part of the putamen (Lang and Lozano, 1998a, Lang and Lozano, 1998b). Dopamine signaling from the SNc to the putamen is thought to have a facilitatory effect upon movement by modulating pathways that link the basal ganglia and cortical motor areas (Alexander et al., 1986). Our observation also replicates other fMRI studies that observed decreased motor activation of the putamen in patients with low UPDRS scores (Holden et al., 2006; Prodoehl et al., 2010; Spraker et al., 2010), all of whom had used a ROI approach to increase the statistical sensitivity for detecting changes in the putamen.

The M1 underactivity observed during motor execution contralateral to the affected hand is likely to reflect the impaired basal ganglia-thalamo-cortical drive that is suggested to produce slowed motor output (Contreras-Vidal and Stelmach, 1995; Moroney et al., 2008). Inappropriate force scaling and bradykinesia may also reflect inadequate activation of motor cortical and spinal cord centers due to reduced dopamine levels (Cutsuridis, 2011). This could manifest as smaller, less forceful, and slower movements in Parkinson's disease patients compared to controls, with a more irregular or ‘noisy’ pattern of finger movements during task performance (e.g. (Mazzoni et al., 2007; Stelmach et al., 1989; Teasdale et al., 1990). Although plausible, we view this less likely, as the key-press requirements during motor execution were identical for patients and controls. It's possible, however, that biomechanical differences in key-press action between patients and controls exist that cannot be determined through key-press data logging alone. While our data possess accurate timing, we do not have any measure of the force used to execute the key-presses that might help to better explain M1 underactivity during affected hand motor execution.

We observed a relative underactivity in the anterior cerebellum / cerebellar vermis which is somehow in contrast to previous fMRI studies that frequently demonstrated cerebellar overactivity in patients with Parkinson's disease during the performance of upper limb movements, as shown for example during internally or externally paced simple finger movements (Cerasa et al., 2006; Rascol et al., 1997; Yu et al., 2007), sequential finger movements (Catalan et al., 1999; Wu and Hallett, 2005), bimanual two-hand coordinated tasks (Wu et al., 2010), motor timing (Jahanshahi et al., 2010), or simultaneous performance of two different motor tasks (Wu and Hallett, 2008). Critically, our study cohort differs in comparison to these previous studies in two ways: (i) our Parkinson's disease patients were early stage UPDRS III (score 16 ± 6; range 7–28), whilst other studies used more advanced Parkinson's disease patients (UPDRS III score 26 ± 3; range 13–34: used as examples here: (Catalan et al., 1999; Cerasa et al., 2006; Rascol et al., 1997; Wu and Hallett, 2005, Wu and Hallett, 2008; Wu et al., 2010; Yu et al., 2007)). Disease stage offers a plausible explanation for these differences to previous research, since later stage Parkinson's disease patients typically show more advanced tremor and/or dyskinesia leading to increased cerebellar activation due to sensory re-afference and error-signal processing; (ii) our PD patients were unmedicated, while PD patients in the fore mentioned studies were typically in an OFF medicated state. Studies in which dopaminergic medication had to be interrupted to achieve OFF-states are hampered by the fact that dopamine agonist drugs have long half-lives, reported as ranging from 3 to 27 h (Jankovic and Aguilar, 2008), although reports suggest the motor effects to last as long as 7 days (Turjanski et al., 1993). This may induce “wearing off” phenomena (Jankovic and Aguilar, 2008) and likely place neuronal circuits in an ‘unusual state’ that is either hyper-responsive or hypo-responsive compared to normal cortical functioning. Hence, it is likely that both the direction and the level of observed activation differences may be affected by disease stage (please refer to (Wu and Hallett, 2013) for a hypothetical model of functional changes accompanying the progression of Parkinson's disease). This aspect of cerebellar underactivity in early Parkinson's disease patients would benefit from further research to elucidate whether task differences or influences in disease stage offer the best explanation for the differences in reported cerebellar activity. In more generals terms cerebellar underactivity may also be explained, at least partially, by reciprocal anatomical connections and interactions between the basal ganglia and the cerebellum (Bostan et al., 2010), along with sensorimotor cortical areas. Bostan et al. (2010) found that the subthalamic nucleus (STN) has a disynaptic topographically organized projection to the cerebellar cortex by way of the pontine nuclei. Most of the STN neurons projecting to Crus II posterior are located in its associative territory, which receives input from the frontal eye fields and regions of the prefrontal cortex. In contrast, most of the STN neurons that project to the hemispheric expansion of lobule VIIB locate in sensorimotor territory, which receives input from the M1 and pre-motor areas. These results suggest that STN-cerebellar connections are involved in the integration of basal ganglia and cerebellar functions, both in motor and non-motor domains (please see for review (Bostan et al., 2010; Wu and Hallett, 2013)).

#### 4.2.2 Motor planning phase

The main effects during the motor planning phase in the within-group analyses are highly consistent with previous literature reported in the context of motor planning processes (Cunnington et al., 2002; Elsinger et al., 2006; Jahanshahi et al., 1995; Jenkins et al., 2000; Weeks et al., 2001), including activation in fronto-parietal regions (i.e. the posterior aspect of the DLPFC (BA9), and Brodmann area 8 including the frontal eye fields, the superior parietal lobe and precuneus (BA5 and BA7) and the supra-marginal gyrus (BA40)), mesial and lateral PMC, contralateral M1, anterior putamen / anterior insula, and lateral cerebellum during *FREE*_PLAN_ in the controls. The Parkinson's disease patients showed a similar cortical activation pattern, and in addition a pronounced activation of the DLPFC (observable bilaterally in *FREE*_PLAN_ and more lateralized in *REACT*_PLAN_ ipsilateral to the affected and contralateral to the non-affected hand, respectively). Additional activation in the rostral basal ganglia (caudate nucleus) was not found. A relative overactivity in the Parkinson's disease patients was evidenced in between-group analyses in the DLPFC ipsilateral to the affected hand and contralateral to the non-affected hand, along with relative overactivity in the ipsilateral anterior cerebellum / cerebellar vermis (during *FREE*_PLAN_ of the NON-AFFECTED HAND) and the precuneus (only during *REACT*_PLAN_). Additionally, ROI analyses complemented these observations, by highlighting a significantly lower percent signal change in the SMA in PD patients compared to healthy controls in both *FREE*_PLAN_, and *REACT*_PLAN_, with a similar tendency in the pre-SMA.

Relative overactivity of the DLPFC in early de novo Parkinson's disease patients is an interesting finding that is suggestive of a compensatory mechanism taking place during the motor planning phase. Anatomically, the DLPFC is densely interconnected with multiple motor areas, including M1 (Miyachi et al., 2005), pre-SMA & PMC (Lu et al., 1994; Tanji and Hoshi, 2008), and the cerebellum (Middleton and Strick, 2001). Despite these anatomical connections to motor cortical regions and despite its involvement in self-generated movements (Boecker et al., 2008; Francois-Brosseau et al., 2009; Jenkins et al., 2000), the role of the DLPFC in self-initiated action has been described as one contributing primarily to attentional or working memory processes and task performance supervision, rather than to movement per se (Wiese et al., 2005). Its specialized role for “attentional–cognitive” processes is supported by the demonstration of increased DLPFC activation when cognitive demands are increased by complexity, or when there is a need to integrate multiple sources of information (Hoshi et al., 2005; Jueptner et al., 1997; Miller and Cohen, 2001; Tanji and Hoshi, 2008; Yamasaki et al., 2002).

Parkinson's disease patients are known to show impairments in executive control, for instance in tasks involving internal action initiation (Michely et al., 2012). In line with the suggested role of the DLPFC for executive control processes, neuroimaging studies have indicated altered DLPFC activation in Parkinson's disease patients compared to healthy controls using PET (Jahanshahi et al., 1995). Moreover, Parkinson's disease patients with cognitive impairment were shown to have a selective underactivity in the DLPFC compared to Parkinson's disease patients without cognitive impairment (and healthy controls) during the manipulation, but not the retrieval, of information within working memory (Lewis et al., 2003). Conversely, fMRI studies have shown increased DLPFC activity during performance of Tower-of-London (Monchi et al., 2004; Monchi et al., 2007) and spatial working memory (Cools et al., 2001) tasks. Similarly, relatively enhanced DLPFC activation was found during sequence learning (Mentis et al., 2003) and motor-sequence retrieval (Nakamura et al., 2001). Such relative overactivity of the DLPFC (along with the PMC, posterior parietal cortex, and precuneus) have been discussed as being part of a so-called “retrieval network” in Parkinson's disease that acts to compensate disease induced network disruption (Nakamura et al., 2001). This interpretation fits with our current results of relative DLPFC and precuneus overactivity in early stage unmedicated Parkinson's disease patients, as compared to healthy controls. Similar to our study, untreated de novo Parkinson's disease patients showed significantly increased task-related activation during a visuospatial working memory task in the left DLPFC (and trend-wise in the right DLPFC), along with the left caudate nucleus, and the left inferior parietal cortex (Trujillo et al., 2015). Additionally, by assessing the functional connectivity of bilateral DLPFC and the effective connectivity within fronto-parietal and fronto-striatal networks, these authors were able to reveal that the left and right DLPFC of Parkinson's disease patients was less strongly connected functionally with prefrontal regions, the precuneus, and the insula during task performance (Trujillo et al., 2015). Inferring that the reduced functional connectivity was likely mediated by disease-related changes, such as the striatal dopamine depletion, may underscore the important role of dopamine in orchestrating connectivity between areas during task performance.

In humans, cerebellar damage is known to cause defects in planning and working memory (Schmahmann and Sherman, 1998). It is thus intriguing that we also found a relative overactivity in the Parkinson's disease patients in the cerebellum / cerebellar vermis during motor planning, while the same area was found to be underactive during motor execution. Hence, findings are suggestive of a compensatory mechanism (in an area with a pathological impairment in Parkinson's disease). It was shown recently (Gao et al., 2018) in mice that had to plan a future directional movement, that transient perturbations of the fastigial nucleus disrupted “subsequent correct responses without hampering movement execution”. Their findings indicated that a cortico-cerebellar loop generates preparatory activity necessary for planning correct motor responses. These finding are very well in line with the observed overactivity in the DLPFC during motor planning in early stage Parkinson's disease. In fact, cerebellar overactivity was also confirmed in additional ROI-analyses. As said, these ROIS were derived from previous imaging studies as being typically affected in Parkinson's disease, notably as an area of overactivity. Our findings add to this general picture, but by demonstrating for the first time that cerebellar overactivity may, at least in early clinical stages, represent a compensatory phenomenon during the planning and conceptualization phase of forthcoming movements. This overactivity may be considered as a mechanism meant to overcome Parkinson's disease pathology, along with the overactivity in DLPFC, in line with Gao and co-workers (Gao et al., 2018). That such compensation was in place is supported by the successful performance of the various components of the task, despite the proven pathology according to DaTSCAN™ scans / clinical assessments.

### 4.3 Limitations

While this study offers several advantages over previous research by providing (i) information about the planning and execution of a well-trained automated movement sequence in de novo Parkinson's disease patients, (ii) two experimental groups with similar performance characteristics, (iii) and being one of the largest fMRI based motor study cohorts in Parkinson's disease patients with a sufficient participant number for robust statistical analysis (Desmond and Glover, 2002), there are some limitations to acknowledge: We attempted to temporally disentangle motor planning from motor execution, however, fMRI allows no clear and complete separation between two intrinsically non-jittered phases of action. Furthermore, having electromyography or video-based data recording of hand movements during task performance would have helped to identify even subtle differences in motor output that could not be captured with the current methods (recoding of button-presses) and would have helped to clarify some of the differences in cortical activation between patients and controls. Finally, novel MRI acquisition schemes like multiband fMRI go along with a higher temporal resolution, allowing a more refined analysis of the underlying physiological and pathophysiological signal changes during motor planning and execution.

## 5 Conclusions

By disentangling the phases of planning and execution, we provide a more refined picture of motor control processes in Parkinson's disease patients. Pertinent to clinical practitioners, this imaging study extends previous fMRI-based literature in Parkinson's disease by disambiguating the relative changes in activation during the planning and execution of freely initiated and reactive action using a finger movement task. Parametric whole brain analysis and ROI based analyses in essence corroborate previous reports of region-specific disruptions due to dopaminergic depletion that become evident during motor execution, but more importantly offer a more refined and novel indication of compensatory mechanisms and changes in neuronal activation that occur in individuals with de novo Parkinson's disease during motor planning. As this reflects a compensatory mechanism during a stage where subsequent motor actions are conceptualized, this offers “another piece in the puzzle towards” an improved pathophysiological understanding in Parkinson's disease. How and whether such data may be used for intervention strategies that focus on the aspect of motor planning is open to debate.

## Appendix A Supplementary data

Supplementary material 1 Image 1

Supplementary material 2 Image 2

## References

[bb0005] Alexander G.E., DeLong M.R., Strick P.L. (1986). Parallel organization of functionally segregated circuits linking basal ganglia and cortex. Annu. Rev. Neurosci..

[bb0010] Ashburner J., Friston K.J. (2005). Unified segmentation. Neuroimage.

[bb0015] Avanzino L., Pelosin E., Martino D., Abbruzzese G. (2013). Motor timing deficits in sequential movements in Parkinson disease are related to action planning: a motor imagery study. PLoS One.

[bb0020] Avecillas-Chasin J.M., Rascon-Ramirez F., Barcia J.A. (2016). Tractographical model of the cortico-basal ganglia and corticothalamic connections: improving our understanding of deep brain stimulation. Clin. Anat..

[bb0025] Benecke R., Rothwell J.C., Dick J.P., Day B.L., Marsden C.D. (1986). Performance of simultaneous movements in patients with Parkinson's disease. Brain.

[bb0030] Benecke R., Rothwell J.C., Dick J.P., Day B.L., Marsden C.D. (1987). Simple and complex movements off and on treatment in patients with Parkinson's disease. J. Neurol. Neurosurg. Psychiatry.

[bb0035] Boecker H., Jankowski J., Ditter P., Scheef L. (2008). A role of the basal ganglia and midbrain nuclei for initiation of motor sequences. Neuroimage.

[bb0040] Bostan A.C., Dum R.P., Strick P.L. (2010). The basal ganglia communicate with the cerebellum. Proc. Natl. Acad. Sci. U. S. A..

[bb0045] Brett M., Anton J., Valabregue R., Poline J. (2002). Region of interest analysis using an SPM toolbox. Neuroimage.

[bb0050] Catalan M.J., Ishii K., Honda M., Samii A., Hallett M. (1999). A PET study of sequential finger movements of varying length in patients with Parkinson's disease. Brain.

[bb0055] Cerasa A., Hagberg G.E., Peppe A., Bianciardi M., Gioia M.C., Costa A., Castriota-Scanderbeg A., Caltagirone C., Sabatini U. (2006). Functional changes in the activity of cerebellum and frontostriatal regions during externally and internally timed movement in Parkinson's disease. Brain Res. Bull..

[bb0060] Contreras-Vidal J.L., Stelmach G.E. (1995). A neural model of basal ganglia-thalamocortical relations in normal and parkinsonian movement. Biol. Cybern..

[bb0065] Cools R., Barker R.A., Sahakian B.J., Robbins T.W. (2001). Enhanced or impaired cognitive function in Parkinson's disease as a function of dopaminergic medication and task demands. Cereb. Cortex.

[bb0070] Cunnington R., Egan G.F., O'Sullivan J.D., Hughes A.J., Bradshaw J.L., Colebatch J.G. (2001). Motor imagery in Parkinson's disease: a PET study. Mov. Disord..

[bb0075] Cunnington R., Windischberger C., Deecke L., Moser E. (2002). The preparation and execution of self-initiated and externally-triggered movement: a study of event-related fMRI. Neuroimage.

[bb0080] Cutsuridis V. (2011). Origins of a repetitive and co-contractive biphasic pattern of muscle activation in Parkinson's disease. Neural Netw..

[bb0085] Deiber M.P., Honda M., Ibanez V., Sadato N., Hallett M. (1999). Mesial motor areas in self-initiated versus externally triggered movements examined with fMRI: effect of movement type and rate. J. Neurophysiol..

[bb0090] Desmond J.E., Glover G.H. (2002). Estimating sample size in functional MRI (fMRI) neuroimaging studies: statistical power analyses. J. Neurosci. Methods.

[bb0095] Dickstein R., Deutsch J.E. (2007). Motor imagery in physical therapist practice. Phys. Ther..

[bb0100] Doyon J. (2008). Motor sequence learning and movement disorders. Curr. Opin. Neurol..

[bb0105] Eickhoff S.B., Stephan K.E., Mohlberg H., Grefkes C., Fink G.R., Amunts K., Zilles K. (2005). A new SPM toolbox for combining probabilistic cytoarchitectonic maps and functional imaging data. Neuroimage.

[bb0110] Eickhoff S.B., Heim S., Zilles K., Amunts K. (2006). Testing anatomically specified hypotheses in functional imaging using cytoarchitectonic maps. Neuroimage.

[bb0115] Eickhoff S.B., Paus T., Caspers S., Grosbras M.H., Evans A.C., Zilles K., Amunts K. (2007). Assignment of functional activations to probabilistic cytoarchitectonic areas revisited. Neuroimage.

[bb0120] Elsinger C.L., Harrington D.L., Rao S.M. (2006). From preparation to online control: reappraisal of neural circuitry mediating internally generated and externally guided actions. Neuroimage.

[bb0125] Fama R., Sullivan E.V. (2002). Motor sequencing in Parkinson's disease: relationship to executive function and motor rigidity. Cortex.

[bb0130] Francois-Brosseau F.E., Martinu K., Strafella A.P., Petrides M., Simard F., Monchi O. (2009). Basal ganglia and frontal involvement in self-generated and externally-triggered finger movements in the dominant and non-dominant hand. Eur. J. Neurosci..

[bb0135] Gao Z., Davis C., Thomas A.M., Economo M.N., Abrego A.M., Svoboda K., De Zeeuw C.I., Li N. (2018). A cortico-cerebellar loop for motor planning. Nature.

[bb0140] Georgiou N., Bradshaw J.L., Iansek R., Phillips J.G., Mattingley J.B., Bradshaw J.A. (1994). Reduction in external cues and movement sequencing in Parkinson's disease. J. Neurol. Neurosurg. Psychiatry.

[bb0145] Harrington D.L., Haaland K.Y. (1991). Sequencing in Parkinson's disease. Abnormalities in programming and controlling movement. Brain.

[bb0150] Hautzinger M. (1991). The Beck depression inventory in clinical practice. Nervenarzt.

[bb0155] Hautzinger M., Bailer M., Worall H., Keller F. (1994). Beck-Depressions-Inventar (BDI). Bearbeitung der deutschen Ausgabe.

[bb0160] Helmich R.C., de Lange F.P., Bloem B.R., Toni I. (2007). Cerebral compensation during motor imagery in Parkinson's disease. Neuropsychologia.

[bb0165] Henson R., Frackowiak R.S.J., Friston K.J., Frith C.D., Dolan R.J., Price C.J., Zeki S. (2004). Analysis of fMRI time series: linear time- invariant models, event-related fMRI, and optimal experimental design. Human Brain Function.

[bb0170] Herz D.M., Eickhoff S.B., Lokkegaard A., Siebner H.R. (2014). Functional neuroimaging of motor control in Parkinson's disease: a meta-analysis. Hum. Brain Mapp..

[bb0175] Hoehn M.M., Yahr M.D. (1967). Parkinsonism: onset, progression and mortality. Neurology.

[bb0180] Holden A., Wilman A., Wieler M., Martin W.R. (2006). Basal ganglia activation in Parkinson's disease. Parkinsonism Relat. Disord..

[bb0185] Hoshi E., Tremblay L., Feger J., Carras P.L., Strick P.L. (2005). The cerebellum communicates with the basal ganglia. Nat. Neurosci..

[bb0190] Jahanshahi M., Jenkins I.H., Brown R.G., Marsden C.D., Passingham R.E., Brooks D.J. (1995). Self-initiated versus externally triggered movements. I. An investigation using measurement of regional cerebral blood flow with PET and movement-related potentials in normal and Parkinson's disease subjects. Brain.

[bb0195] Jahanshahi M., Jones C.R., Zijlmans J., Katzenschlager R., Lee L., Quinn N., Frith C.D., Lees A.J. (2010). Dopaminergic modulation of striato-frontal connectivity during motor timing in Parkinson's disease. Brain.

[bb0200] Jankovic J., Aguilar L.G. (2008). Current approaches to the treatment of Parkinson's disease. Neuropsychiatr. Dis. Treat..

[bb0205] Jankowski J., Scheef L., Huppe C., Boecker H. (2009). Distinct striatal regions for planning and executing novel and automated movement sequences. Neuroimage.

[bb0210] Jankowski J., Paus S., Scheef L., Bewersdorff M., Schild H.H., Klockgether T., Boecker H. (2013). Abnormal movement preparation in task-specific focal hand dystonia. PLoS One.

[bb0215] Jenkins I.H., Jahanshahi M., Jueptner M., Passingham R.E., Brooks D.J. (2000). Self-initiated versus externally triggered movements. II. The effect of movement predictability on regional cerebral blood flow. Brain.

[bb0220] Jueptner M., Stephan K.M., Frith C.D., Brooks D.J., Frackowiak R.S., Passingham R.E. (1997). Anatomy of motor learning. I. Frontal cortex and attention to action. J. Neurophysiol..

[bb0225] Jung W.H., Jang J.H., Park J.W., Kim E., Goo E.H., Im O.S., Kwon J.S. (2014). Unravelling the intrinsic functional organization of the human striatum: a parcellation and connectivity study based on resting-state FMRI. PLoS One.

[bb0230] Lang A., Fahn S., TL, M. (1989). Assessment of Parkinson's disease. Quantification of Neurological Deficit.

[bb0235] Lang A.E., Lozano A.M. (1998). Parkinson's disease. First of two parts. N. Engl. J. Med..

[bb0240] Lang A.E., Lozano A.M. (1998). Parkinson's disease. Second of two parts. N. Engl. J. Med..

[bb0245] Lees A.J., Hardy J., Revesz T. (2009). Parkinson's disease. Lancet.

[bb0250] Lewis S.J., Dove A., Robbins T.W., Barker R.A., Owen A.M. (2003). Cognitive impairments in early Parkinson's disease are accompanied by reductions in activity in frontostriatal neural circuitry. J. Neurosci..

[bb0255] Lu M.T., Preston J.B., Strick P.L. (1994). Interconnections between the prefrontal cortex and the premotor areas in the frontal lobe. J. Comp. Neurol..

[bb0260] Ma J., Ma S., Zou H., Zhang Y., Chan P., Ye Z. (2018). Impaired serial ordering in nondemented patients with mild Parkinson's disease. PLoS One.

[bb0265] Mahoney M., Avener M. (1977). Psychology of the elite athlete: an exploratory study. Cogn. Ther. Res..

[bb0270] Maillet A., Thobois S., Fraix V., Redoute J., Le Bars D., Lavenne F., Derost P., Durif F., Bloem B.R., Krack P., Pollak P., Debu B. (2015). Neural substrates of levodopa-responsive gait disorders and freezing in advanced Parkinson's disease: a kinesthetic imagery approach. Hum. Brain Mapp..

[bb0275] Maldjian J.A., Laurienti P.J., Kraft R.A., Burdette J.H. (2003). An automated method for neuroanatomic and cytoarchitectonic atlas-based interrogation of fMRI data sets. Neuroimage.

[bb0280] Maldjian J.A., Laurienti P.J., Burdette J.H. (2004). Precentral gyrus discrepancy in electronic versions of the Talairach atlas. Neuroimage.

[bb0285] Marsden C.D. (1987). What do the basal ganglia tell premotor cortical areas?. CIBA Found. Symp..

[bb0290] Marsden C.D. (1989). Slowness of movement in Parkinson's disease. Mov. Disord..

[bb0295] Martin J.A., Karnath H.O., Himmelbach M. (2015). Revisiting the cortical system for peripheral reaching at the parieto-occipital junction. Cortex.

[bb0300] Mazzoni P., Hristova A., Krakauer J.W. (2007). Why don't we move faster? Parkinson's disease, movement vigor, and implicit motivation. J. Neurosci..

[bb0305] Mentis M.J., Dhawan V., Feigin A., Delalot D., Zgaljardic D., Edwards C., Eidelberg D. (2003). Early stage Parkinson's disease patients and normal volunteers: comparative mechanisms of sequence learning. Hum. Brain Mapp..

[bb0310] Michely J., Barbe M.T., Hoffstaedter F., Timmermann L., Eickhoff S.B., Fink G.R., Grefkes C. (2012). Differential effects of dopaminergic medication on basic motor performance and executive functions in Parkinson's disease. Neuropsychologia.

[bb0315] Middleton F.A., Strick P.L. (2001). Cerebellar projections to the prefrontal cortex of the primate. J. Neurosci..

[bb0320] Miller E.K., Cohen J.D. (2001). An integrative theory of prefrontal cortex function. Annu. Rev. Neurosci..

[bb0325] Miyachi S., Lu X., Inoue S., Iwasaki T., Koike S., Nambu A., Takada M. (2005). Organization of multisynaptic inputs from prefrontal cortex to primary motor cortex as revealed by retrograde transneuronal transport of rabies virus. J. Neurosci..

[bb0330] Mochizuki-Kawai H., Mochizuki S., Kawamura M. (2010). A flexible sequential learning deficit in patients with Parkinson's disease: a 2 x 8 button-press task. Exp. Brain Res..

[bb0335] Monchi O., Petrides M., Doyon J., Postuma R.B., Worsley K., Dagher A. (2004). Neural bases of set-shifting deficits in Parkinson's disease. J. Neurosci..

[bb0340] Monchi O., Petrides M., Mejia-Constain B., Strafella A.P. (2007). Cortical activity in Parkinson's disease during executive processing depends on striatal involvement. Brain.

[bb0345] Moroney R., Heida C., Geelen J. (2008). Increased bradykinesia in Parkinson's disease with increased movement complexity: elbow flexion-extension movements. J. Comput. Neurosci..

[bb0350] Nakamura T., Ghilardi M.F., Mentis M., Dhawan V., Fukuda M., Hacking A., Moeller J.R., Ghez C., Eidelberg D. (2001). Functional networks in motor sequence learning: abnormal topographies in Parkinson's disease. Hum. Brain Mapp..

[bb0355] Oldfield R.C. (1971). The assessment and analysis of handedness: the Edinburgh inventory. Neuropsychologia.

[bb0360] Pieruccini-Faria F., Jones J.A., Almeida Q.J. (2014). Motor planning in Parkinson's disease patients experiencing freezing of gait: the influence of cognitive load when approaching obstacles. Brain Cogn..

[bb0365] Pieruccini-Faria F., Jones J.A., Almeida Q.J. (2016). Insight into dopamine-dependent planning deficits in Parkinson's disease: a sharing of cognitive & sensory resources. Neuroscience.

[bb0370] Prodoehl J., Spraker M., Corcos D., Comella C., Vaillancourt D. (2010). Blood oxygenation level-dependent activation in basal ganglia nuclei relates to specific symptoms in de novo Parkinson's disease. Mov. Disord..

[bb0375] Rascol O., Sabatini U., Fabre N., Brefel C., Loubinoux I., Celsis P., Senard J.M., Montastruc J.L., Chollet F. (1997). The ipsilateral cerebellar hemisphere is overactive during hand movements in akinetic parkinsonian patients. Brain.

[bb0380] Sadato N., Ibanez V., Campbell G., Deiber M.P., Le Bihan D., Hallett M. (1997). Frequency-dependent changes of regional cerebral blood flow during finger movements: functional MRI compared to PET. J. Cereb. Blood Flow Metab..

[bb0385] Samuel M., Ceballos-Baumann A.O., Boecker H., Brooks D.J. (2001). Motor imagery in normal subjects and Parkinson's disease patients: an H215O PET study. Neuroreport.

[bb0390] Schmahmann J.D., Sherman J.C. (1998). The cerebellar cognitive affective syndrome. Brain.

[bb0395] Sladky R., Friston K.J., Trostl J., Cunnington R., Moser E., Windischberger C. (2011). Slice-timing effects and their correction in functional MRI. Neuroimage.

[bb0400] Spraker M.B., Prodoehl J., Corcos D.M., Comella C.L., Vaillancourt D.E. (2010). Basal ganglia hypoactivity during grip force in drug naive Parkinson's disease. Hum. Brain Mapp..

[bb0405] Stelmach G.E., Teasdale N., Phillips J., Worringham C.J. (1989). Force production characteristics in Parkinson's disease. Exp. Brain Res..

[bb0410] Tanji J., Hoshi E. (2008). Role of the lateral prefrontal cortex in executive behavioral control. Physiol. Rev..

[bb0415] Teasdale N., Phillips J., Stelmach G.E. (1990). Temporal movement control in patients with Parkinson's disease. J. Neurol. Neurosurg. Psychiatry.

[bb0420] Thobois S., Dominey P.F., Decety J., Pollak P.P., Gregoire M.C., Le Bars P.D., Broussolle E. (2000). Motor imagery in normal subjects and in asymmetrical Parkinson's disease: a PET study. Neurology.

[bb0425] Tremblay P.L., Bedard M.A., Langlois D., Blanchet P.J., Lemay M., Parent M. (2010). Movement chunking during sequence learning is a dopamine-dependant process: a study conducted in Parkinson's disease. Exp. Brain Res..

[bb0430] Trujillo J.P., Gerrits N.J., Veltman D.J., Berendse H.W., van der Werf Y.D., van den Heuvel O.A. (2015). Reduced neural connectivity but increased task-related activity during working memory in de novo Parkinson patients. Hum. Brain Mapp..

[bb0435] Turjanski N., Fernandez W., Lees A.J. (1993). The effects of acute levodopa withdrawal on motor performance and dopaminergic receptor sensitivity in patients with Parkinson's disease. J. Neurol. Neurosurg. Psychiatry.

[bb0440] Vriezen E.R., Moscovitch M. (1990). Memory for temporal order and conditional associative-learning in patients with Parkinson's disease. Neuropsychologia.

[bb0445] Weeks R.A., Honda M., Catalan M.J., Hallett M. (2001). Comparison of auditory, somatosensory, and visually instructed and internally generated finger movements: a PET study. Neuroimage.

[bb0450] Wiese H., Stude P., Nebel K., Forsting M., de Greiff A. (2005). Prefrontal cortex activity in self-initiated movements is condition-specific, but not movement-related. Neuroimage.

[bb0455] Wu T., Hallett M. (2005). A functional MRI study of automatic movements in patients with Parkinson's disease. Brain.

[bb0460] Wu T., Hallett M. (2008). Neural correlates of dual task performance in patients with Parkinson's disease. J. Neurol. Neurosurg. Psychiatry.

[bb0465] Wu T., Hallett M. (2013). The cerebellum in Parkinson's disease. Brain.

[bb0470] Wu T., Wang L., Hallett M., Li K., Chan P. (2010). Neural correlates of bimanual anti-phase and in-phase movements in Parkinson's disease. Brain.

[bb0475] Yamasaki H., LaBar K.S., McCarthy G. (2002). Dissociable prefrontal brain systems for attention and emotion. Proc. Natl. Acad. Sci. U. S. A..

[bb0480] Yu H., Sternad D., Corcos D.M., Vaillancourt D.E. (2007). Role of hyperactive cerebellum and motor cortex in Parkinson's disease. Neuroimage.

