# Supplementary material for: Disentangling motor planning and motor execution in unmedicated de novo Parkinson's disease patients: An fMRI study
Source: Neuroimage Clin. 2019 Mar 19;22:101784. doi: 10.1016/j.nicl.2019.101784 (PMC6438987; doi:10.1016/j.nicl.2019.101784)
Supplement: Supplementary file 1 — Supplementary material 1 [file mmc1.docx]

Supplementary Material document 1 – SPM12 statistics for Within-group activations (shown in Figure 2) for the preparation and execution of action, compared to REST, in healthy control participants and de novo Parkinson’s disease patients.

Figure 2 A – FREE_PLAN_ Affected hand – Controls.

|  |  |  |  |  |  | MNI |  |  |
| --- | --- | --- | --- | --- | --- | --- | --- | --- |
| Cortical | Regions | BA | K | T | Z | x | y | z |
| Right | Supplementary Motor Area | 6 | 1968 | 9.65 | Inf | 3 | 2 | 59 |
| Left | Superior Frontal Gyrus | 6 |  | 9.61 | Inf | -24 | -1 | 56 |
| Right | Lateral Occipital Cortex | 7 |  | 9.15 | Inf | 12 | -70 | 56 |
| Right | Insular Cortex | 48 | 119 | 7.73 | 7.41 | 36 | 17 | 5 |
| Right |  |  |  | 5.57 | 5.45 | 54 | 8 | 14 |
| Left | Frontal Operculum Cortex | 48 | 84 | 7.67 | 7.36 | -36 | 14 | 5 |
| Left | Superior Parietal Lobule | 40 | 479 | 7.53 | 7.24 | -30 | -46 | 38 |
| Left |  |  |  | 7.38 | 7.1 | -33 | -52 | 53 |
| Left |  |  |  | 6.89 | 6.66 | -12 | -70 | 53 |
| Left | Middle Frontal Gyrus | 45 | 34 | 7.34 | 7.06 | -42 | 32 | 35 |
| Right | Dorso-lateral prefrontal cortex | 46 | 151 | 7.05 | 6.8 | 39 | 44 | 26 |
| Right |  |  |  | 5.55 | 5.42 | 36 | 29 | 32 |
| Left | Temporal Occipital Fusiform Gyrus | 37 | 65 | 6.17 | 6 | -30 | -52 | -31 |
| Left | Precentral Gyrus | 44 | 25 | 6.00 | 5.84 | -51 | 5 | 29 |
| Right | Lateral Occipital Cortex | 37 | 73 | 5.61 | 5.48 | 48 | -67 | -1 |
| Right |  |  |  | 5.31 | 5.2 | 33 | -76 | 11 |
| Right | Temporal Occipital Fusiform Gyrus | 37 | 13 | 5.38 | 5.26 | 39 | -52 | -31 |
| Right | Precentral Gyrus | 44 | 15 | 5.30 | 5.19 | 51 | 5 | 32 |

Figure 2 B – FREE_PLAN_ Affected hand– Parkinson’s disease patients.

|  |  |  |  |  |  | MNI |  |  |
| --- | --- | --- | --- | --- | --- | --- | --- | --- |
| Cortical | Regions | BA | K | T | Z | x | y | z |
| Right | Superior Frontal Gyrus | 6 | 1856 | 9.63 | Inf | 24 | -7 | 62 |
| Right |  | 6 |  | 9.34 | Inf | 24 | 2 | 56 |
|  | Paracingulate gyrus | 32 |  | 8.71 | Inf | 0 | 8 | 50 |
| Left | Middle Frontal Gyrus | 46 | 161 | 8.63 | Inf | -33 | 35 | 26 |
| Left |  | 46 |  | 5.98 | 5.82 | -27 | 56 | 20 |
| Right | Temporal Occipital Fusiform Gyrus | 37 | 112 | 7.82 | 7.49 | 33 | -52 | -31 |
| Right |  |  |  | 6.97 | 6.73 | 42 | -55 | -34 |
| Right |  |  |  | 5.28 | 5.17 | 27 | -67 | -22 |
| Left | Temporal Occipital Fusiform Gyrus | 37 | 198 | 7.74 | 7.42 | -33 | -49 | -34 |
| Left |  |  |  | 6.51 | 6.31 | -33 | -52 | -46 |
| Left | Lateral Occipital Cortex | 7 | 149 | 7.47 | 7.18 | -15 | -67 | 53 |
| Left | Superior Pareital Lobe | 40 | 127 | 7.46 | 7.17 | -39 | -43 | 47 |
| Right | Lateral Occipital Cortex | 7 | 86 | 7.32 | 7.05 | 15 | -64 | 50 |
| Right |  |  |  | 6.32 | 6.14 | 15 | -64 | 62 |
| Right | Dorso-lateral prefrontal cortex | 46 | 128 | 6.42 | 6.23 | 33 | 44 | 23 |
| Right |  |  |  | 6.06 | 5.9 | 33 | 32 | 35 |
| Right |  |  |  | 5.55 | 5.43 | 30 | 56 | 8 |
| Right | Insular cortex | 48 | 44 | 6.40 | 6.21 | 33 | 20 | 5 |
| Left |  | 48 | 91 | 6.07 | 5.91 | -24 | 5 | 2 |
| Left |  |  |  | 5.71 | 5.58 | -33 | 17 | 5 |
| Left | Opercular cortex | 48 | 12 | 5.53 | 5.4 | -51 | 8 | -1 |
| Right | Premotor cortex and Supplementary Motor Cortex | 6 | 15 | 5.20 | 5.1 | 24 | 5 | 2 |

Figure 2 C – FREE_PLAN_ Non-affected hand – Controls.

|  |  |  |  |  |  | MNI |  |  |
| --- | --- | --- | --- | --- | --- | --- | --- | --- |
| Cortical | Regions | BA | K | T | Z | x | y | z |
| Left | Precentral Gyrus | 56 | 1825 | 10.06 | Inf | -33 | -19 | 56 |
| Left | Superior Frontal Gyrus | 6 |  | 9.43 | Inf | -24 | -1 | 56 |
| Left | Supplementary Motor Area | 6 |  | 9.20 | Inf | -6 | 2 | 53 |
| Right | Lateral Occipital Cortex | 7 | 606 | 8.84 | Inf | 12 | -70 | 56 |
| Right |  | 40 |  | 7.69 | 7.37 | 42 | -34 | 41 |
| Right |  | 7 |  | 7.58 | 7.27 | 21 | -67 | 44 |
| Right | Frontal Operculum Cortex | 48 | 120 | 7.66 | 7.35 | 39 | 14 | 5 |
| Right | Precentral Gyrus | 6 |  | 5.36 | 5.25 | 54 | 8 | 14 |
| Left | Insular Cortex | 48 | 82 | 7.57 | 7.27 | -30 | 14 | 5 |
| Left | Middle Frontal Gyrus | 45 | 28 | 7.06 | 6.81 | -42 | 32 | 35 |
| Right | Dorso-lateral prefrontal cortex | 46 | 140 | 6.75 | 6.53 | 39 | 41 | 26 |
| Left | Precentral Gyrus | 44 | 28 | 6.11 | 5.95 | -51 | 5 | 29 |
| Right | Lateral Occipital Cortex | 37 | 50 | 5.41 | 5.29 | 48 | -64 | -4 |
| Right | Middle occipital gyrus | 19 |  | 4.88 | 4.79 | 42 | -76 | 5 |
| Right | Precentral Gyrus | 44 | 8 | 5.32 | 5.21 | 48 | 5 | 32 |
| Right | Middle occipital gyrus | 19 | 17 | 5.30 | 5.19 | 9 | -52 | -16 |

Figure 2 D – FREE_PLAN_ Non-affected hand– Parkinson’s disease patients.

|  |  |  |  |  |  | MNI |  |  |
| --- | --- | --- | --- | --- | --- | --- | --- | --- |
| Cortical | Regions | BA | K | T | Z | x | y | z |
| Left | Superior frontal gyrus | 6 | 1923 | 11.16 | Inf | -21 | -7 | 65 |
| Left | Postcentral gyrus | 40 |  | 9.66 | Inf | -36 | -37 | 47 |
| Left | Dorsal anterior cingulate cortex | 32 |  | 9.08 | Inf | -3 | 8 | 50 |
| Left | Dorso-lateral prefrontal cortex | 46 | 223 | 9.12 | Inf | -33 | 35 | 26 |
| Left |  | 46 |  | 5.80 | 5.66 | -30 | 47 | 20 |
| Left |  | 46 |  | 5.29 | 5.18 | -30 | 56 | 17 |
| Right | Fusiform gyrus | 37 | 237 | 8.92 | Inf | 33 | -52 | -31 |
| Right |  | 37 |  | 7.97 | 7.61 | 18 | -52 | -22 |
| Right | Middle occipital gyrus | 19 |  | 6.29 | 6.11 | 24 | -64 | -22 |
| Left | Lateral Occipital Cortex | 7 | 161 | 8.56 | Inf | -15 | -67 | 53 |
| Right | Superior Parietal Lobe | 40 | 207 | 8.16 | 7.78 | 36 | -43 | 44 |
| Right |  | 40 |  | 6.33 | 6.15 | 51 | -34 | 44 |
| Left | Temporal Occipital Fusiform Gyrus | 37 | 125 | 7.58 | 7.28 | -39 | -55 | -34 |
| Left |  |  |  | 5.06 | 4.96 | -33 | -58 | -43 |
| Right | Lateral Occipital Cortex | 7 | 70 | 7.10 | 6.85 | 15 | -67 | 53 |
| Right | Insular Cortex | 48 | 51 | 6.85 | 6.62 | 33 | 20 | 5 |
| Left | Central Opercular Cortex | 48 | 24 | 6.82 | 6.59 | -51 | 8 | -1 |
| Right | Dorso-lateral prefrontal cortex | 46 | 172 | 6.64 | 6.43 | 33 | 32 | 35 |
| Right |  |  |  | 6.55 | 6.35 | 33 | 47 | 20 |
| Left | Insular Cortex | 48 | 41 | 5.89 | 5.74 | -30 | 20 | 5 |
| Left |  | 48 |  | 5.01 | 4.92 | -18 | 14 | 2 |

Figure 2 E – FREE_MOTOR_ Affected hand – Controls.

|  |  |  |  |  |  | MNI |  |  |
| --- | --- | --- | --- | --- | --- | --- | --- | --- |
| Cortical | Regions | BA | K | T | Z | x | y | z |
| Left | Temporal Occipital Fusiform Gyrus | 37 | 1671 | 16.79 | Inf | -18 | -49 | -22 |
| Left |  | 18 |  | 16.77 | Inf | -6 | -61 | -13 |
| Right | Postcentral Gyrus | 4 | 2414 | 15.06 | Inf | 39 | -28 | 59 |
| Right | Precentral Gyrus | 6 |  | 14.36 | Inf | 30 | -13 | 65 |
| Right | Parietal Operculum Cortex | 48 |  | 11.96 | Inf | 48 | -19 | 20 |
| Left | Postcentral Gyrus | 2 | 697 | 9.09 | Inf | -51 | -28 | 47 |
| Left | Postcentral Gyrus | 48 |  | 8.00 | 7.64 | -60 | -19 | 29 |
| Left | Postcentral Gyrus | 40 |  | 7.82 | 7.49 | -36 | -37 | 53 |
| Right | Basal Ganglia | 48 | 593 | 8.95 | Inf | 24 | -10 | 2 |
| Right |  | 48 |  | 8.47 | Inf | 30 | -16 | 8 |
| Left | Premotor cortex and Supplementary Motor Cortex | 6 | 128 | 7.68 | 7.36 | -30 | -7 | 62 |
| Left | Insular Cortex | 48 | 88 | 7.37 | 7.08 | -36 | -1 | 11 |
| Left |  |  | 42 | 6.16 | 5.99 | -21 | -10 | 5 |
| Left | Precentral Gyrus | 6 | 49 | 6.12 | 5.95 | -57 | 5 | 32 |
| Left |  | 6 |  | 4.88 | 4.79 | -54 | -1 | 44 |

Figure 2 F – FREE_MOTOR_ Affected hand– Parkinson’s disease patients.

|  |  |  |  |  |  | MNI |  |  |
| --- | --- | --- | --- | --- | --- | --- | --- | --- |
| Cortical | Regions | BA | K | T | Z | x | y | z |
| Left | Fusiform gyrus | 37 | 1844 | 13.68 | Inf | -30 | -55 | -25 |
| Left | Middle occipital gyrus | 19 |  | 13.64 | Inf | -24 | -61 | -22 |
| Right | Middle Frontal Gyrus | 3 | 2537 | 13.31 | Inf | 54 | -22 | 47 |
| Right | Postcentral Gyrus | 4 |  | 13.08 | Inf | 42 | -28 | 59 |
| Right |  | 4 |  | 12.87 | Inf | 39 | -19 | 65 |
| Right | Precentral Gyrus | 48 | 391 | 8.32 | Inf | 60 | 8 | 5 |
| Right |  | 48 |  | 7.57 | 7.26 | 24 | 2 | 5 |
| Right |  | 48 |  | 7.28 | 7.01 | 54 | 14 | -4 |
| Left | Central Opercular Cortex | 48 | 300 | 8.22 | 7.83 | -51 | 5 | -1 |
| Left |  | 48 |  | 7.62 | 7.31 | -57 | 5 | 11 |
| Left |  | 48 |  | 7.09 | 6.84 | -39 | 2 | 2 |
| Left | Supramarginal Gyrus | 48 | 502 | 7.69 | 7.37 | -63 | -25 | 29 |
| Left |  | 48 |  | 7.05 | 6.81 | -60 | -22 | 20 |
| Left | Postcentral Gyrus | 40 |  | 6.85 | 6.62 | -36 | -37 | 50 |

Figure 2 G – FREE_MOTOR_ Non-affected hand – Controls.

|  |  |  |  |  |  | MNI |  |  |
| --- | --- | --- | --- | --- | --- | --- | --- | --- |
| Cortical | Regions | BA | K | T | Z | x | y | z |
| Right | Temporal Occipital Fusiform Gyrus | 37 | 1627 | 15.88 | Inf | 21 | -49 | -25 |
| Right |  | 18 |  | 15.32 | Inf | 6 | -58 | -13 |
| Left | Postcentral Gyrus | 2 | 2896 | 14.62 | Inf | -48 | -28 | 50 |
| Left | Premotor cortex and Supplementary Motor Cortex | 6 |  | 14.42 | Inf | -33 | -19 | 62 |
| Left | Precentral Gyrus | 3 |  | 13.89 | Inf | -39 | -34 | 56 |
| Right | Postcentral Gyrus | 2 | 625 | 8.52 | Inf | 42 | -31 | 44 |
| Right | Postcentral Gyrus | 48 |  | 7.36 | 7.08 | 63 | -19 | 23 |
| Right |  | 2 |  | 6.88 | 6.65 | 42 | -40 | 59 |
| Left | Precentral Gyrus | 6 | 62 | 7.05 | 6.8 | -57 | 5 | 32 |
| Right | Precentral Gyrus | 6 | 50 | 6.67 | 6.46 | 30 | -10 | 65 |
| Right | Insular Cortex | 48 | 46 | 6.60 | 6.4 | 39 | -1 | 11 |
| Right | Precentral Gyrus | 6 | 90 | 6.43 | 6.24 | 57 | 8 | 32 |
| Right |  | 6 |  | 5.75 | 5.61 | 57 | 11 | 11 |
| Right | Inferior Frontal Gyrus, pars opercularis | 48 |  | 5.49 | 5.36 | 57 | 14 | -1 |
| Right | Basal Ganglia | 48 | 46 | 6.29 | 6.11 | 24 | -7 | 5 |
|  |  | 23 | 30 | 6.11 | 5.94 | 0 | -31 | -43 |

Figure 2 H – FREE_MOTOR_ Non-affected hand– Parkinson’s disease patients.

|  |  |  |  |  |  | MNI |  |  |
| --- | --- | --- | --- | --- | --- | --- | --- | --- |
| Cortical | Regions | BA | K | T | Z | x | y | z |
| Right | Fusiform gyrus | 37 | 1220 | 14.47 | Inf | 24 | -55 | -22 |
| Right | Lingual Gyrus | 18 |  | 12.27 | Inf | 9 | -58 | -13 |
| Left | Precentral Gyrus | 6 | 2310 | 13.78 | Inf | -33 | -22 | 65 |
| Left |  | 3 |  | 11.42 | Inf | -39 | -34 | 53 |
| Left |  | 2 |  | 10.82 | Inf | -33 | -43 | 63 |
| Left | Middle occipital gyrus | 19 | 364 | 7.88 | 7.54 | -24 | -64 | -22 |
| Left | Fusiform gyrus | 37 |  | 7.82 | 7.48 | -42 | -55 | -31 |
| Left |  | 37 |  | 7.51 | 7.21 | -33 | -55 | -25 |
| Left | Insular Cortex | 48 | 382 | 7.34 | 7.06 | -30 | -1 | 8 |
| Left |  | 48 |  | 7.26 | 6.99 | -57 | 5 | 11 |
| Left |  |  |  | 7.10 | 6.85 | -24 | -7 | 11 |
| Right | Postcentral Gyrus | 3 | 297 | 7.24 | 6.97 | 54 | -22 | 47 |
| Right | Central Opercular Cortex | 48 |  | 6.24 | 6.06 | 60 | -16 | 20 |
| Right | Superior Pareital Lobule | 40 |  | 6.19 | 6.01 | 39 | -43 | 59 |
| Right | Inferior Frontal Gyrus, pars opercularis | 48 | 59 | 6.57 | 6.36 | 60 | 11 | 5 |
| Right | Basal Ganglia | 48 | 39 | 6.14 | 5.97 | 21 | 5 | 5 |
| Right | Premotor cortex and Supplementary Motor Cortex | 6 | 19 | 5.64 | 5.51 | 27 | -7 | 65 |

Figure 2 I – REACT_PLAN_ Affected hand – Controls.

|  |  |  |  |  |  | MNI |  |  |
| --- | --- | --- | --- | --- | --- | --- | --- | --- |
| Cortical | Regions | BA | K | T | Z | x | y | z |
| Right | Supplementary Motor Area | 6 | 176 | 7.56 | 7.26 | 3 | 2 | 59 |
| Left | Supplementary Motor Area | 6 |  | 6.78 | 6.55 | -6 | 2 | 56 |
| Right | Occipital Cortex | 18 | 24 | 6.91 | 6.68 | 21 | -94 | -4 |
| Right | Precentral Gyrus | 6 | 207 | 6.80 | 6.58 | 33 | -13 | 56 |
| Right | Postcentral Gyrus | 4 |  | 5.92 | 5.77 | 33 | -25 | 50 |
| Right | Frontal Operculum Cortex | 48 | 96 | 6.58 | 6.38 | 39 | 17 | 5 |
| Right | Frontal Orbital Cortex | 47 |  | 6.14 | 5.97 | 33 | 29 | 2 |
| Left | Frontal Operculum Cortex | 48 | 41 | 6.11 | 5.94 | -39 | 17 | 8 |
| Left | Supramarginal Gyrus (posterior) | 40 | 14 | 5.19 | 5.08 | -30 | -49 | 38 |

Figure 2 J – REACT_PLAN_ Affected hand– Parkinson’s disease patients.

|  |  |  |  |  |  | MNI |  |  |
| --- | --- | --- | --- | --- | --- | --- | --- | --- |
| Cortical | Regions | BA | K | T | Z | x | y | z |
| Left | Paracingulate Gyrus | 32 | 1056 | 8.72 | Inf | -9 | 11 | 47 |
| Right | Premotor cortex and Supplementary Motor Cortex | 6 |  | 8.55 | Inf | 27 | -7 | 62 |
| Right |  | 6 |  | 8.48 | Inf | 3 | 5 | 50 |
| Left | Middle Frontal Gyrus | 48 | 89 | 8.47 | Inf | -30 | 32 | 23 |
| Left |  | 48 | 82 | 7.06 | 6.81 | -27 | 23 | 5 |
| Left |  | 48 |  | 5.41 | 5.29 | -24 | 5 | 2 |
| Right | Insular Cortex | 48 | 49 | 6.72 | 6.5 | 33 | 23 | 5 |
| Left | Fusiform gyrus | 37 | 51 | 6.20 | 6.03 | -33 | -49 | -33 |
| Left |  | 37 |  | 5.39 | 5.28 | -42 | -55 | -31 |
| Left |  | 48 | 17 | 6.17 | 6 | -51 | 5 | -1 |
| Right | Fusiform gyrus | 37 | 32 | 5.95 | 5.79 | 45 | -58 | -31 |
| Right |  | 37 |  | 5.58 | 5.46 | 33 | -52 | -31 |
| Left | Superior Parietal Lobule | 40 | 32 | 5.92 | 5.77 | -39 | -40 | 47 |
| Right | Lateral Occipital Cortex | 7 | 16 | 5.72 | 5.58 | 15 | -64 | 50 |
| Right | Dorso-lateral prefrontal cortex | 46 | 12 | 5.50 | 5.38 | 33 | 35 | 32 |
| Right | Middle occipital gyrus | 19 | 5 | 5.41 | 5.3 | 24 | -67 | -22 |
| Left | Lateral Occipital Cortex | 7 | 14 | 5.29 | 5.18 | -15 | -64 | 53 |
| Left |  | 7 |  | 5.10 | 5 | -21 | -67 | 59 |

Figure 2 K – REACT_PLAN_ Non-affected hand – Controls.

|  |  |  |  |  |  | MNI |  |  |
| --- | --- | --- | --- | --- | --- | --- | --- | --- |
| Cortical | Regions | BA | K | T | Z | x | y | z |
| Left | Precentral Gyrus | 4 | 376 | 10.21 | Inf | -33 | -19 | 56 |
| Left | Postcentral Gyrus | 3 |  | 4.93 | 4.84 | -51 | -22 | 44 |
| Left | Supplementary Motor Area | 6 | 213 | 8.46 | Inf | -6 | 2 | 53 |
| Right |  | 6 |  | 7.48 | 7.19 | 3 | 2 | 59 |
| Right | Dorsal anterior cingulate cortex | 32 |  | 5.63 | 5.5 | 12 | 8 | 44 |
| Right | Insular Cortex | 48 | 88 | 6.91 | 6.68 | 39 | 17 | 5 |
| Right | Occipital Cortex | 18 | 18 | 6.56 | 6.36 | 21 | -94 | -4 |
| Left | Frontal Operculum Cortex | 48 | 37 | 5.71 | 5.57 | -36 | 17 | 5 |
| Left | Superior Parietal Lobule | 40 | 26 | 5.44 | 5.32 | -33 | -52 | 56 |
| Left | Superior Parietal Lobule | 40 | 21 | 5.22 | 5.11 | -36 | -37 | 41 |
| Left |  | 40 |  | 5.00 | 4.91 | -30 | -43 | 38 |

Figure 2 L – REACT_PLAN_ Non-affected hand– Parkinson’s disease patients.

|  |  |  |  |  |  | MNI |  |  |
| --- | --- | --- | --- | --- | --- | --- | --- | --- |
| Cortical | Regions | BA | K | T | Z | x | y | z |
| Left | Superior Frontal Gyrus | 6 | 914 | 9.62 | Inf | -18 | -7 | 65 |
| Left | Dorsal anterior cingulate cortex | 32 |  | 8.80 | Inf | -3 | 8 | 50 |
| Left |  | 32 |  | 8.74 | Inf | -12 | 8 | 44 |
| Left | Middle Frontal Gyrus | 48 | 91 | 9.03 | Inf | -30 | 32 | 26 |
| Left | Dorso-lateral prefrontal cortex | 46 |  | 4.75 | 4.67 | -27 | 47 | 20 |
| Left | Inferior Frontal Gyrus, pars opercularis | 47 | 75 | 7.80 | 7.47 | -30 | 26 | 5 |
| Right | Frontal Operculum Cortex | 48 | 58 | 6.93 | 6.7 | 33 | 23 | 11 |
| Right | Superior Parietal Lobule | 40 | 43 | 6.27 | 6.09 | 36 | -46 | 44 |
| Right | Fusiform gyrus | 37 | 74 | 6.23 | 6.05 | 33 | -49 | -31 |
| Right |  | 37 |  | 6.08 | 5.92 | 18 | -52 | -22 |
| Right | Middle occipital gyrus | 19 |  | 5.21 | 5.11 | 24 | -64 | -22 |
| Right | Premotor cortex and Supplementary Motor Cortex | 6 | 63 | 6.22 | 6.05 | 21 | -1 | 50 |
| Right | Premotor cortex and Supplementary Motor Cortex | 6 |  | 5.22 | 5.12 | 24 | -4 | 62 |
| Left | Central Opercular Cortex | 48 | 12 | 5.90 | 5.75 | -51 | 8 | -1 |
| Left | Lateral Occipital Cortex | 7 | 27 | 5.45 | 5.33 | -21 | -64 | 59 |
| Left |  | 7 |  | 5.43 | 5.31 | -15 | -64 | 53 |

Figure 2 M – REACT_MOTOR_ Affected hand – Controls.

|  |  |  |  |  |  | MNI |  |  |
| --- | --- | --- | --- | --- | --- | --- | --- | --- |
| Cortical | Regions | BA | K | T | Z | x | y | z |
| Left | Temporal Occipital Fusiform Gyrus | 37 | 1845 | 16.50 | Inf | -18 | -49 | -22 |
| Left | Lingual Gyrus | 18 |  | 16.48 | Inf | -9 | -61 | -16 |
| Right | Postcentral Gyrus | 3 | 2750 | 15.63 | Inf | 36 | -28 | 56 |
| Right | Precentral Gyrus | 6 |  | 14.89 | Inf | 30 | -13 | 65 |
| Right | Postcentral Gyrus | 3 |  | 13.67 | Inf | 51 | -22 | 47 |
| Right | Central Opercular Cortex | 48 | 749 | 10.03 | Inf | 42 | -1 | 11 |
| Right |  |  |  | 8.53 | Inf | 24 | -7 | 2 |
| Right |  |  |  | 8.39 | Inf | 15 | -16 | 5 |
| Left | Postcentral Gyrus | 2 | 774 | 9.26 | Inf | -48 | -28 | 47 |
| Left | Postcentral Gyrus | 48 |  | 9.04 | Inf | -57 | -19 | 26 |
| Left | Premotor cortex and Supplementary Motor Cortex | 6 |  | 9.02 | Inf | -54 | -22 | 41 |
| Left | Insular Cortex | 48 | 221 | 8.20 | 7.81 | -36 | -1 | 11 |
| Left | Precentral Gyrus | 6 |  | 7.81 | 7.48 | -57 | 5 | 32 |
| Left | Central Opercular Cortex | 48 |  | 7.31 | 7.03 | -45 | 2 | 8 |
| Left | Precentral Gyrus | 6 | 154 | 8.14 | 7.76 | -30 | -10 | 62 |
| Left | Basal Ganglia |  | 27 | 5.71 | 5.57 | -15 | -13 | -1 |

Figure 2 N – REACT_MOTOR_ Affected hand– Parkinson’s disease patients.

|  |  |  |  |  |  | MNI |  |  |
| --- | --- | --- | --- | --- | --- | --- | --- | --- |
| Cortical | Regions | BA | K | T | Z | x | y | z |
| Left | Fusiform gyrus | 37 | 2031 | 14.98 | Inf | -30 | -55 | -25 |
| Left | Middle occipital gyrus | 19 |  | 14.43 | Inf | -24 | -61 | -22 |
| Left | Lateral Occipital Cortex | 7 |  | 13.41 | Inf | -15 | -61 | 49 |
| Right | Postcentral Gyrus | 4 | 3539 | 13.82 | Inf | 54 | -19 | 47 |
| Right |  | 4 |  | 13.41 | Inf | 42 | -25 | 59 |
| Right |  | 4 |  | 13.31 | Inf | 39 | -19 | 65 |
| Left | Precentral Gyrus | 48 | 375 | 9.27 | Inf | -57 | 5 | 11 |
| Left |  | 48 |  | 8.73 | Inf | -51 | 8 | -1 |
| Left | Premotor cortex and Supplementary Motor Cortex | 6 |  | 8.33 | Inf | -57 | 5 | 26 |
| Left | Supramarginal Gyrus (anterior) | 2 | 752 | 9.26 | Inf | -60 | -28 | 35 |
| Left | Postcentral Gyrus | 40 |  | 8.49 | Inf | -36 | -37 | 50 |
| Left |  | 42 |  | 7.91 | 7.56 | -63 | -22 | 20 |

Figure 2 O – REACT_MOTOR_ Non-affected hand – Controls.

|  |  |  |  |  |  | MNI |  |  |
| --- | --- | --- | --- | --- | --- | --- | --- | --- |
| Cortical | Regions | BA | K | T | Z | x | y | z |
| Right | Lingual Gryus | 18 | 1900 | 16.36 | Inf | 9 | -58 | -16 |
| Right | Temporal Occipital Fusiform Gyrus | 37 |  | 16.35 | Inf | 21 | -52 | -22 |
| Left | Precentral Gyrus | 6 | 3750 | 15.36 | Inf | -33 | -19 | 62 |
| Left |  | 3 |  | 15.21 | Inf | -36 | -34 | 53 |
| Left | Postcentral Gyrus | 2 |  | 15.17 | Inf | -48 | -28 | 50 |
| Right | Supramarginal Gyrus | 2 | 795 | 9.07 | Inf | 42 | -31 | 41 |
| Right | Parietal operculum Cortex | 48 |  | 8.64 | Inf | 60 | -22 | 20 |
| Right |  | 48 |  | 8.14 | 7.76 | 57 | -31 | 23 |
| Right | Inferior Frontal Gyrus, pars opercularis | 6 | 385 | 7.36 | 7.08 | 57 | 11 | 11 |
| Right | Central Opercular Cortex | 48 |  | 7.22 | 6.96 | 42 | -1 | 11 |
| Right | Precentral Gyrus | 6 |  | 6.75 | 6.53 | 60 | 8 | 32 |
| Left | Superior Parietal Lobule | 7 | 134 | 7.25 | 6.98 | -27 | -55 | 55 |

Figure 2 P – REACT_MOTOR_ Non-affected hand– Parkinson’s disease patients.

|  |  |  |  |  |  | MNI |  |  |
| --- | --- | --- | --- | --- | --- | --- | --- | --- |
| Cortical | Regions | BA | K | T | Z | x | y | z |
| Right | Temporal Occipital Fusiform Gyrus | 37 | 2166 | 16.99 | Inf | 24 | -55 | -22 |
| Right |  | 18 |  | 14.51 | Inf | 9 | -58 | -13 |
| Left | Precentral Gyrus | 6 | 4355 | 16.09 | Inf | -33 | -19 | 65 |
| Left |  | 6 |  | 15.65 | Inf | -42 | -25 | 59 |
| Left |  | 6 |  | 13.55 | Inf | -21 | -10 | 68 |
| Right | Inferior Frontal Gyrus, pars opercularis | 48 | 455 | 8.71 | Inf | 60 | 11 | 5 |
| Right | Precentral Gyrus | 6 |  | 8.04 | 7.68 | 60 | 8 | 23 |
| Right | Inferior Frontal Gyrus, pars opercularis | 38 |  | 8.00 | 7.65 | 54 | 14 | -4 |
| Right | Postcentral Gyrus | 48 | 811 | 8.49 | Inf | 57 | -16 | 29 |
| Right |  | 48 |  | 8.44 | Inf | 60 | -16 | 20 |
| Right | Postcentral Gyrus | 3 |  | 8.24 | 7.84 | 54 | -22 | 47 |
| Left | Intracalcarine Cortex | 17 | 72 | 5.66 | 5.53 | -12 | -67 | 5 |
| Left |  | 17 |  | 5.17 | 5.06 | -6 | -76 | 11 |
