# Supplementary material for: Disentangling motor planning and motor execution in unmedicated de novo Parkinson's disease patients: An fMRI study
Source: Neuroimage Clin. 2019 Mar 19;22:101784. doi: 10.1016/j.nicl.2019.101784 (PMC6438987; doi:10.1016/j.nicl.2019.101784)
Supplement: Supplementary file 2 — Supplementary material 2 [file mmc2.docx]

Supplementary Material document 2 – SPM12 statistics for Between-group activation comparisons (shown in Figure 3) for the preparation and execution of action, in healthy control participants and de novo Parkinson’s disease patients.

| Figure 3 A - FREE_PLAN_ – Affected  Controls > PD | |  |  |  |  |  |  |  |
| --- | --- | --- | --- | --- | --- | --- | --- | --- |
|  |  |  |  |  |  | MNI |  |  |
| Cortical | Regions | BA | K | T | Z | x | y | z |
| Right | Middle occipital gyrus | 19 | 86 | 4.85 | 4.76 | 36 | -76 | 14 |
|  |  |  |  |  |  |  |  |  |

| Figure 3 B - FREE_PLAN_ – Affected  PD > Controls | |  |  |  |  |  |  |  |
| --- | --- | --- | --- | --- | --- | --- | --- | --- |
|  |  |  |  |  |  | MNI |  |  |
| Cortical | Regions | BA | K | T | Z | x | y | z |
| Left | Dorso-lateral prefrontal cortex | 9 | 99 | 5.9 | 5.75 | -30 | 32 | 23 |
|  |  |  |  |  |  |  |  |  |

| Figure 3 C - FREE_PLAN_ – Non affected  Controls > PD | |  |  |  |  |  |  |  |
| --- | --- | --- | --- | --- | --- | --- | --- | --- |
|  |  |  |  |  |  | MNI |  |  |
| Cortical | Regions | BA | K | T | Z | x | y | z |
| Left | Superior Parietal Lobule | 7 | 25 | 4.3 | 4.24 | -27 | -52 | 56 |
| Left | pars Triangularis | 45 | 24 | 4.04 | 3.99 | -51 | 20 | 26 |
|  |  |  |  |  |  |  |  |  |
| Right | Middle occipital gyrus | 19 | 50 | 4.12 | 4.06 | 39 | -76 | 8 |

| Figure 3 D - FREE_PLAN_ – Non affected  PD > Controls | |  |  |  |  |  |  |  |
| --- | --- | --- | --- | --- | --- | --- | --- | --- |
|  |  |  |  |  |  | MNI |  |  |
| Cortical | Regions | BA | K | T | Z | x | y | z |
| Left | Dorso-lateral prefrontal cortex | 9 | 111 | 6.43 | 6.23 | -30 | 32 | 23 |
|  |  |  |  |  |  |  |  |  |
| Right | Cerebelum Lobule 8 |  | 112 | 5.31 | 5.2 | 15 | -70 | -40 |
|  |  |  |  | 3.39 | 3.36 | 30 | -58 | -52 |
| Right | Cerebelum Lobule 6 |  | 87 | 4.9 | 4.82 | 24 | -67 | -19 |
|  |  |  |  |  |  |  |  |  |
| Right | Putamen (Posterior) / Pallidum | 48 | 84 | 4.43 | 4.36 | 27 | -19 | -4 |
|  |  | 48 |  | 3.93 | 3.88 | 36 | -10 | -10 |
|  |  | 48 |  | 3.75 | 3.71 | 39 | 2 | -19 |
|  |  |  |  |  |  |  |  |  |

| Figure 3 E - FREE_MOTOR_ – Affected  Controls > PD | |  |  |  |  |  |  |  |
| --- | --- | --- | --- | --- | --- | --- | --- | --- |
|  |  |  |  |  |  | MNI |  |  |
| Cortical | Regions | BA | K | T | Z | x | y | z |
| Left | Cerebelum Lobule 4-5 |  | 317 | 6.04 | 5.88 | -15 | -49 | -22 |
|  |  |  |  | 5.62 | 5.49 | -6 | -61 | -13 |
|  |  |  |  | 3.88 | 3.83 | -6 | -61 | -31 |
| Right | Putamen (Posterior) / Thalamus (motor) |  | 91 | 5.15 | 5.05 | 21 | -10 | -4 |
|  | Putamen (Posterior) | 48 |  | 4.57 | 4.49 | 30 | -13 | 8 |
|  | Thalamus |  |  | 3.34 | 3.31 | 18 | -19 | 11 |
| Right | Primary Motor cortex (Precentral gyrus) | 4 | 306 | 5.06 | 4.96 | 33 | -25 | 53 |
|  |  |  |  | 4.68 | 4.6 | 33 | -13 | 68 |
|  |  |  |  | 3.87 | 3.83 | 42 | -34 | 44 |
| Right | Middle Temporal Gyrus | 21 | 152 | 4.67 | 4.59 | 48 | -55 | 14 |
|  |  | 42 |  | 3.84 | 3.8 | 54 | -37 | 17 |
|  |  | 42 |  | 3.47 | 3.44 | 63 | -43 | 20 |

| Figure 3 F - FREE_MOTOR_ – Affected  PD > Controls | |  |  |  |  |  |  |  |
| --- | --- | --- | --- | --- | --- | --- | --- | --- |
|  |  |  |  |  |  | MNI |  |  |
| Cortical | Regions | BA | K | T | Z | x | y | z |
| Left | Dorso-lateral prefrontal cortex | 9 | 36 | 4.37 | 4.31 | -24 | 32 | 23 |
|  |  |  |  |  |  |  |  |  |

| Figure 3 G - FREE_MOTOR_ – Non affected  Controls > PD | |  |  |  |  |  |  |  |
| --- | --- | --- | --- | --- | --- | --- | --- | --- |
|  |  |  |  |  |  | MNI |  |  |
| Cortical | Regions | BA | K | T | Z | x | y | z |
| Left | Putamen (Posterior) / Pallidum |  | 88 | 4.2 | 4.14 | -24 | -25 | 8 |
|  | Putamen (Posterior) | 48 |  | 4.18 | 4.12 | -30 | -13 | 2 |
|  | Thalamus |  |  | 3.86 | 3.82 | -12 | -13 | -1 |
|  | Brain Stem / anterior Cerebellum – midline |  | 236 | 4.42 | 4.36 | 3 | -34 | -46 |
|  |  |  |  | 4.41 | 4.34 | 9 | -28 | -37 |
|  |  |  |  | 4.39 | 4.32 | 6 | -55 | -19 |

| Figure 3 H - FREE_MOTOR_ – Non affected  PD > Controls | |  |  |  |  |  |  |  |
| --- | --- | --- | --- | --- | --- | --- | --- | --- |
|  |  |  |  |  |  | MNI |  |  |
| Cortical | Regions | BA | K | T | Z | x | y | z |
| Left | Dorso-lateral prefrontal cortex | 9 | 9 | 3.61 | 3.57 | -27 | 32 | 23 |
| Left | Ventricle WM |  | 10 | 3.58 | 3.54 | -33 | -31 | 26 |
|  |  |  | 7 | 3.57 | 3.53 | -27 | -19 | 29 |
|  |  |  |  |  |  |  |  |  |
| Right | Ventricle WM |  | 13 | 3.53 | 3.5 | 24 | -25 | 26 |

| Figure 3 I - REACT_PLAN_ – Affected  Controls > PD | |  |  |  |  |  |  |  |
| --- | --- | --- | --- | --- | --- | --- | --- | --- |
|  |  |  |  |  |  | MNI |  |  |
| Cortical | Regions | BA | K | T | Z | x | y | z |
| Left | Mid orbital gyrus – Frontal lobe | 10 | 30 | 4.77 | 4.68 | -6 | 50 | -4 |
|  | Superior medial gyrus – Frontal lobe | 10 |  | 4.41 | 4.34 | -3 | 53 | 8 |
|  |  |  |  |  |  |  |  |  |
|  | Inferior frontal gyrus – (pars Triangularis) | 45 | 43 | 4.21 | 4.15 | -54 | 17 | 29 |
|  |  |  |  |  |  |  |  |  |
| Right | Middle Temporal lobe | 21 | 56 | 4.14 | 4.08 | 45 | -52 | 14 |
|  | Middle Temporal lobe | 21 |  | 4.09 | 4.04 | 45 | -46 | 8 |
|  |  |  |  |  |  |  |  |  |

| Figure 3 J - REACT_PLAN_ – Affected  PD > Controls | |  |  |  |  |  |  |  |
| --- | --- | --- | --- | --- | --- | --- | --- | --- |
|  |  |  |  |  |  | MNI |  |  |
| Cortical | Regions | BA | K | T | Z | x | y | z |
| Left | Dorso-lateral prefrontal cortex | 46 | 138 | 7.05 | 6.8 | -30 | 32 | 23 |
|  |  |  |  |  |  |  |  |  |
| Right | Cerebelum Lobule 8 |  | 177 | 4.73 | 4.65 | 12 | -73 | -43 |
| Left | Cerebelum Lobule 9 |  |  | 4.08 | 4.02 | -6 | -58 | -52 |
| Right | Cerebelum Lobule 8 |  |  | 4.03 | 3.98 | 30 | -58 | -52 |
|  |  |  |  |  |  |  |  |  |
| Left | Cerebelum |  | 91 | 4.57 | 4.49 | -24 | -43 | -40 |
|  | Cerebelum Lobule 7 Crus1 |  |  | 4.22 | 4.16 | -39 | -55 | -40 |
|  | Cerebelum Lobule 8 |  |  | 3.57 | 3.53 | -30 | -58 | -46 |
|  |  |  |  |  |  |  |  |  |
| Right | Visual cortex area 3 | 18 | 73 | 4 | 3.95 | 6 | -73 | 23 |
| Right | Cuneus / Posterior parietal cortex | 18 |  | 3.98 | 3.93 | 3 | -79 | 32 |
| Right | Cuneus | 19 |  | 3.62 | 3.58 | 15 | -70 | 20 |
|  |  |  |  |  |  |  |  |  |

| Figure 3 K - REACT_PLAN_ – Non affected  Controls > PD | |  |  |  |  |  |  |  |
| --- | --- | --- | --- | --- | --- | --- | --- | --- |
|  |  |  |  |  |  | MNI |  |  |
| Cortical | Regions | BA | K | T | Z | x | y | z |
| Left | Inferior Frontal Gyrus (pars orbitalis) | 46 | 21 | 4.6 | 4.53 | -36 | 32 | -10 |
| Left | Middle Frontal Gyrus | 44 | 22 | 4.37 | 4.31 | -36 | 14 | 38 |
| Left | Inferior frontal gyrus – (pars Triangularis) | 45 | 29 | 4.09 | 4.04 | -51 | 20 | 26 |
| Right | Middle Temporal Gyrus | 39 | 20 | 4.03 | 3.98 | 42 | -52 | 14 |

| Figure 3 L - REACT_PLAN_ – Non affected  PD > Controls | |  |  |  |  |  |  |  |
| --- | --- | --- | --- | --- | --- | --- | --- | --- |
|  |  |  |  |  |  | MNI |  |  |
| Cortical | Regions | BA | K | T | Z | x | y | z |
| Left | Dorso-lateral prefrontal cortex | 9 | 201 | 6.92 | 6.69 | -30 | 32 | 23 |
|  |  | 9 |  | 5.43 | 5.31 | -27 | 32 | 32 |
|  |  |  |  | 4.12 | 4.07 | -24 | 47 | 23 |
| Right | Putamen (Posterior) | 20 | 146 | 5.58 | 5.46 | 30 | -22 | -4 |
|  |  | 48 |  | 4.04 | 3.99 | 45 | -16 | -10 |
| Right | Cerebelum Lobule 8 |  | 93 | 5.07 | 4.97 | 15 | -70 | -40 |
|  |  |  |  | 3.59 | 3.55 | 30 | -58 | -52 |
| Right | Cerebelum Lobule 6 |  | 343 | 4.86 | 4.78 | 15 | -73 | 20 |
|  |  |  |  | 4.74 | 4.66 | 6 | -70 | 20 |
|  |  |  |  | 4.63 | 4.56 | -3 | -79 | 26 |

| Figure 3 M - REACT_MOTOR_ – Affected  Controls > PD | |  |  |  |  |  |  |  |
| --- | --- | --- | --- | --- | --- | --- | --- | --- |
|  |  |  |  |  |  | MNI |  |  |
| Cortical | Regions | BA | K | T | Z | x | y | z |
| Right | Putamen (Posterior) | 48 | 111 | 5.61 | 5.48 | 33 | -10 | 5 |
|  | Thalamus |  |  | 4.19 | 4.14 | 12 | -10 | -1 |
|  | Thalamus / Pallidum |  |  | 4.05 | 4 | 24 | -19 | 14 |
|  |  |  |  |  |  |  |  |  |
| Left | Cerebelum Lobule 4-5 |  | 335 | 5.58 | 5.45 | -15 | -49 | -19 |
|  | Cerebelum Lobule 5 |  |  | 5.05 | 4.95 | -6 | -61 | -16 |
|  | Cerebelum Lobule 4-5 |  |  | 4.47 | 4.4 | -21 | -37 | -22 |
|  |  |  |  |  |  |  |  |  |
| Right | Precentral Gyrus | 4 | 230 | 5.45 | 5.33 | 30 | -25 | 53 |
|  | Precentral Gyrus |  |  | 3.4 | 3.37 | 18 | -25 | 50 |
|  | Precentral Gyrus |  |  | 3.32 | 3.29 | 21 | -25 | 68 |
| Right | Middle Temporal Gyrus | 37 | 112 | 4.42 | 4.35 | 54 | -64 | 8 |
|  | Middle Temporal Gyrus | 37 |  | 4.39 | 4.32 | 45 | -61 | -4 |

| Figure 3 N - REACT_MOTOR_ – Affected  PD > Controls | |  |  |  |  |  |  |  |
| --- | --- | --- | --- | --- | --- | --- | --- | --- |
|  |  |  |  |  |  | MNI |  |  |
| Cortical | Regions | BA | K | T | Z | x | y | z |
| Left | Supramarginal gyrus | 39 | 27 | 3.82 | 3.77 | -33 | -43 | 23 |
|  | Supramarginal gyrus | 39 |  | 3.61 | 3.57 | -30 | -55 | 26 |

| Figure 3 O - REACT_MOTOR_ – Non affected  Controls > PD | |  |  |  |  |  |  |  |
| --- | --- | --- | --- | --- | --- | --- | --- | --- |
|  |  |  |  |  |  | MNI |  |  |
| Cortical | Regions | BA | K | T | Z | x | y | z |
| Right | Cerebellum (Anterior) |  | 146 | 4.54 | 4.47 | 12 | -46 | -28 |
| Right | Cerebellar Vermis 4 / 5 |  |  | 4.48 | 4.41 | 3 | -58 | -19 |
|  | Cerebellar Vermis 10 |  |  | 3.74 | 3.7 | 0 | -49 | -28 |

| Figure 3 P - REACT_MOTOR_ – Non affected  PD > Controls | |  |  |  |  |  |  |  |
| --- | --- | --- | --- | --- | --- | --- | --- | --- |
|  |  |  |  |  |  | MNI |  |  |
| Cortical | Regions | BA | K | T | Z | x | y | z |
| Right | Precuneus | 5 | 51 | 4.03 | 3.98 | 3 | -52 | 59 |
| Left | Precuneus | 5 |  | 3.2 | 3.17 | -6 | -40 | 56 |
